# Metabolic reprogramming induced by ketone bodies diminishes pancreatic cancer cachexia

**DOI:** 10.1186/2049-3002-2-18

**Published:** 2014-09-01

**Authors:** Surendra K Shukla, Teklab Gebregiworgis, Vinee Purohit, Nina V Chaika, Venugopal Gunda, Prakash Radhakrishnan, Kamiya Mehla, Iraklis I Pipinos, Robert Powers, Fang Yu, Pankaj K Singh

**Affiliations:** 1 The Eppley Institute for Research in Cancer and Allied Diseases, University of Nebraska Medical Center, Omaha, NE 68198, USA; 2 Department of Chemistry, University of Nebraska—Lincoln, Lincoln, NE 68588, USA; 3 Department of Pathology and Microbiology, University of Nebraska Medical Center, Omaha, NE 68198, USA; 4 Department of Cellular and Integrative Physiology, University of Nebraska Medical Center, Omaha, NE 68198, USA; 5 Department of Surgery, University of Nebraska Medical Center, Omaha, NE 68198, USA; 6 Department of Biostatistics, University of Nebraska Medical Center, Omaha, NE 68198, USA; 7 Department of Biochemistry and Molecular Biology, University of Nebraska Medical Center, Omaha, NE 68198, USA; 8 Department of Genetic Cell Biology and Anatomy, University of Nebraska Medical Center, Omaha, NE 68198, USA

**Keywords:** Pancreatic cancer, Cancer cachexia, Cancer metabolism, Ketone bodies

## Abstract

**Background:**

Aberrant energy metabolism is a hallmark of cancer. To fulfill the increased energy requirements, tumor cells secrete cytokines/factors inducing muscle and fat degradation in cancer patients, a condition known as cancer cachexia. It accounts for nearly 20% of all cancer-related deaths. However, the mechanistic basis of cancer cachexia and therapies targeting cancer cachexia thus far remain elusive. A ketogenic diet, a high-fat and low-carbohydrate diet that elevates circulating levels of ketone bodies (*i.e.*, acetoacetate, β-hydroxybutyrate, and acetone), serves as an alternative energy source. It has also been proposed that a ketogenic diet leads to systemic metabolic changes. Keeping in view the significant role of metabolic alterations in cancer, we hypothesized that a ketogenic diet may diminish glycolytic flux in tumor cells to alleviate cachexia syndrome and, hence, may provide an efficient therapeutic strategy.

**Results:**

We observed reduced glycolytic flux in tumor cells upon treatment with ketone bodies. Ketone bodies also diminished glutamine uptake, overall ATP content, and survival in multiple pancreatic cancer cell lines, while inducing apoptosis. A decrease in levels of c-Myc, a metabolic master regulator, and its recruitment on glycolytic gene promoters, was in part responsible for the metabolic phenotype in tumor cells. Ketone body-induced intracellular metabolomic reprogramming in pancreatic cancer cells also leads to a significantly diminished cachexia in cell line models. Our mouse orthotopic xenograft models further confirmed the effect of a ketogenic diet in diminishing tumor growth and cachexia.

**Conclusions:**

Thus, our studies demonstrate that the cachectic phenotype is in part due to metabolic alterations in tumor cells, which can be reverted by a ketogenic diet, causing reduced tumor growth and inhibition of muscle and body weight loss.

## Background

Pancreatic cancer is the fourth leading cause of cancer-related deaths in the USA [1]. Pancreatic ductal adenocarcinoma (PDAC) accounts for 95% of all pancreatic cancer cases [2]. Despite advances in the understanding of pancreatic cancer biology, effective chemotherapeutic modalities for the treatment of patients remain to be developed. In addition to the aggressive pathogenesis, around 83% of pancreatic cancer patients demonstrate cancer-induced cachexia, which significantly contributes to cancer-related deaths [3]. Thus, inhibition of cachexia along with cancer cell growth may be an effective strategy for the management of pancreatic cancer.

Cachexia, a metabolic syndrome, leads to a loss of muscle weight and the depletion of fat deposits. Although an association of cachexia with various types of cancers has been known for a long time, the molecular mechanism of cancer-induced cachexia is poorly understood [4]. Cachexia is triggered by a large number of tumor and host-derived catabolic factors and pro-inflammatory cytokines such as IL-6, TNFα, and IFN-γ, which lead to changes in host metabolism and energy expenditure [4]. It has been proposed that excessive consumption of glucose by a growing tumor first leads to a depletion of glucose in the blood. At later stages of tumor growth, a depletion of glycogen stores in the liver occurs. Glycogen depletion is followed by muscle degradation and depletion of adipose deposits. All these account for the cachexia syndrome and result in a poor response to chemotherapy, fatigue, and a reduced quality of life for cancer patients [5].

Cancer cells exhibit reprogramming of several metabolic pathways along with multiple genetic, epigenetic, and growth signaling alterations [6,7]. Most cancer cells demonstrate an increase in glucose uptake, a higher rate of glycolysis, and an increase in lactate secretion despite the presence of oxygen, a phenomenon known as the Warburg effect [8]. Aerobic glycolysis plays an important role in rapid cellular growth as it provides several intermediates required for biomass synthesis by routing the carbon flux through the pentose phosphate pathway [9]. The increased conversion of pyruvate into lactate by aerobic glycolysis leads to acidosis in tumor microenvironments that facilitates invasion and metastasis of cancer cells [10]. Aerobic glycolysis is also an energy-inefficient process requiring large amounts of glucose. Correspondingly, tumor cells serve as a glucose sink [11]. Additionally, lactate produced from tumor cells passes to the liver and gets converted to glucose by means of the Cori cycle, another energy-inefficient process [9]. Along with glucose uptake and enhanced aerobic glycolysis, cancer patients also present glucose intolerance and increased hepatic glucose production [12]. An increased requirement for glucose might be the critical stimulus needed for enhanced hepatic glucose production. Tumor cells also have alterations in the metabolism of glutamine, a nitrogen source and arguably the most significant metabolite precursor for tumor cells after glucose [13].

A ketogenic diet is a high-fat and low-carbohydrate diet that leads to elevated circulating levels of ketone bodies (*i.e.*, acetoacetate, β-hydroxybutyrate, and acetone) and an alternative energy source [14]. Ketogenic diets possess anticonvulsant and antiinflammatory activities [15,16]. It has also been proposed that a ketogenic diet treatment results in systemic metabolic changes like increased glucose tolerance, reduced fatty acid synthesis, and weight loss [17]. Keeping in view the significant role of inflammation and metabolic alterations in cancer, a ketogenic diet may provide an efficient therapeutic strategy. Furthermore, most cancer cells lack key mitochondrial enzymes to metabolize ketone bodies and generate ATP, while myocytes and other tissues, including the brain, still retain this ability [18]. Hence, a ketogenic diet may act against the cancer-induced cachexia while causing minimal side effects as previously it has been shown that a 2–7-mM ketone body concentration can be achieved safely without giving rise to clinical acidosis [19,20]. In the present study, we have evaluated anticancerous and anticachectic properties of ketone bodies in cell culture conditions, as well as the effect of a ketogenic diet on tumor burden and cachexia in animal models*.* Furthermore, our studies establish a ketone body-induced metabolomic reprogramming as the mechanism of action of a ketogenic diet against cancer and cancer-induced cachexia.

## Methods

### Cells and reagents

The human pancreatic cancer cell line Capan1, mouse myoblast C2C12, and mouse embryo fibroblast (preadipocyte) 3T3L1 were obtained from American Type Culture Collection (Manassas, VA, USA). S2-013 is a cloned subline of a human pancreatic tumor cell line (SUIT-2) derived from a liver metastasis [21]. All the cell lines were cultured in Dulbecco’s modified Eagle’s medium (DMEM) supplemented with 10% fetal bovine serum, penicillin (100 mg/mL), and streptomycin (100 mg/mL) and incubated at 37°C in a humidified chamber with 5% CO_2_. Sodium-3-hydroxybutyrate, lithium acetoacetate, dihydroethidium (DHE), 3-[4,5-dimethylthiazol-2-yl]-2,5-diphenyltetrazolium bromide (MTT), BCPCF, and *S*-hydroxy butyric acid were purchased from Sigma Chemicals (Sigma-Aldrich, St. Louis, MO, USA).

### Cell viability and caspase 3/7 activity assay

Cell viability was determined by performing MTT assay. Capan1 and S2-013 cells (5 × 10^3^ cells per well) were seeded in 96-well plates for 12 h and then treated with different concentrations of sodium-3-hydroxybutyrate or lithium acetoacetate for 72 h. After treatment, cells were incubated with MTT reagent for 2 h; the resultant formazan crystals were dissolved in dimethyl sulfoxide and the absorbance was recorded at 590 nm. Untreated cells were utilized as a control for the viability assays. Caspase 3/7 activity was determined by utilizing a Promega Caspase-Glo kit (Madison, WI, USA). Capan1 and S2-013 cells (0.6 × 10^6^ cells per well) were seeded in 6-well plates for 12 h and then treated with different concentrations of sodium-3-hydroxybutyrate and lithium acetoacetate for 48 h. Caspase 3/7 activity was then determined as per the manufacturer’s protocol.

### Glucose and glutamine uptake assay

To determine glucose uptake, Capan1 and S2-013 cells (5 × 10^4^ cells per well) were seeded in 24-well plates. After 12 h, cells were treated with multiple concentrations of sodium-3-hydroxybutyrate and lithium acetoacetate for 24 h. After treatment, cells were starved for glucose for 2 h and then incubated for 20 min with 1 μCi [^3^H]-2-deoxyglucose (DG) for a glucose uptake assay. Cells were washed with phosphate-buffered saline (PBS) and lysed with 1% sodium dodecyl sulfate (SDS). The lysates were then subjected to [^3^H] counting by utilizing a scintillation counter. Scintillation counts from cells treated with labeled and excess unlabeled 2-DG were utilized as controls for baseline correction. The results were normalized to the cell counts. For determining glutamine uptake, Capan1 and S2-013 cells (5 × 10^4^ cells per well) were seeded in 24-well plates. After 12 h, cells were treated with solvent control, multiple concentrations of sodium-3-hydroxybutyrate, or lithium acetoacetate for 24 h. Post treatment, cells were starved for glutamine for 2 h and then incubated for 3 min with 1 μCi tritiated Glutamine, l-[3,4-^3^H(N)]. Cells were washed with PBS and lysed in 1% SDS. The lysates were used for [^3^H] counting by utilizing a scintillation counter. Scintillation counts from cells treated with labeled and excess unlabeled glutamine were utilized as controls for baseline correction. The results were normalized to the cell counts.

### Lactate release assay

Capan1 and S2-013 cells (5 × 10^4^ cells per well) were seeded in 24-well plates. After 12 h, cells were treated with indicated concentrations of sodium-3-hydroxybutyrate or lithium acetoacetate for 24 h. The culture supernatants were then utilized for determining lactate release. The assay was performed by utilizing a Lactate Assay Kit (Eton Bioscience Inc., San Diego, CA, USA), as per the manufacturer’s protocol.

### ATP assay

Total ATP level in cells was determined by using an ATP assay kit (Roche, Indianapolis, IN, USA). After 12 h, cells were treated with different concentrations of sodium-3-hydroxybutyrate and lithium acetoacetate for 24 h and ATP level was determined as per the manufacturer’s protocol. ATP level was normalized with total protein concentration.

### Reactive oxygen species assay

Reactive oxygen species level was determined by using oxidation-sensitive fluorescent dye DHE. Capan1 and S2-013 cells (0.1 × 10^6^ cells per well) were seeded in 12-well plates on glass coverslips. After 12 h, cells were treated with solvent control or indicated doses of sodium-3-hydroxybutyrate and lithium acetoacetate for 24 h. Control and treated cells were incubated at 37°C in 2.5 mM DHE containing DMEM. After incubation, cells were washed with cold PBS and fixed with HistoChoice® fixative (Sigma-Aldrich) for 15 min at room temperature. Cells were washed with PBS and the coverslips were mounted onto glass slides using real-mount. Fluorescence intensity per cell was determined by scanning with Zeiss Axiovert 200 M microscope (Oberkochen, Germany) and analyzing the images with SlideBook 5.5 software (Intelligent Imaging Innovations, Inc., Denver, CO, USA).

### Gene expression analysis by qRT-PCR

Total RNA was isolated by utilizing RNeasy columns (Qiagen, Venlo, The Netherlands) as per the manufacturer’s protocol. Total RNA (5 μg) was reverse transcribed by utilizing Verso-cDNA synthesis kit (Thermo Scientific, Pittsburgh, PA, USA) according to the manufacturer’s guidelines. Quantitative reverse transcription polymerase chain reaction (qRT-PCR) was performed with gene-specific primers at 95°C for 10 s and 60°C for 60 s (40 cycles) in 10 μL reaction mix containing 3 μL cDNA, 2 μL primers, and 5 μL SYBR Green Master Mix (Applied Biosystems, Grand Island, NY, USA) using an ABI 7500 thermocycler. Beta-actin was utilized as an internal control. The sequence of different sets of primers used in the study is given in Additional file 1. Quantification was performed with the ΔΔCt method [22].

### Immunoblotting

For immunoblotting, cells were washed twice with cold PBS and lysed in RIPA lysis buffer by incubating at 4°C rotatory shaker for 30 min. Cell debris was removed by centrifugation at 13,000 rpm for 10 min and the supernatant was collected. Protein content was measured by performing Bradford assay. Western blotting was performed as described previously [23]. The membranes were probed with primary antibody against GLUT1 (Abcam, Cambridge, UK), c-Myc-9E10 (Santa Cruz Biotechnology, Dallas, TX, USA), HKII (Cell Signaling Technology, Beverly, MA, USA), and HSP90 (Santa Cruz Biotechnology).

### Luciferase assay

c-Myc-promoter (del1)-luciferase reporter construct was obtained from Addgene (Cambridge, MA, USA) [24]. Cells were transfected with 1 μg of plasmid, and 16 h post transfection, cells were treated with different ketone bodies for 24 h. A synthetic *Renilla* luciferase reporter pRL-TK was utilized as a transfection control. Luciferase activity was determined by utilizing Dual-Luciferase Reporter Assay System (Promega).

### Chromatin immunoprecipitation

For chromatin immunoprecipitation, cells were treated with 20 mM sodium-3-hydroxybutyrate and lithium acetoacetate for 24 h along with solvent control. Chromatin immunoprecipitation was performed by utilizing c-Myc antibody (9E10) as described previously [25]. Mouse IgG was utilized as a control. qPCR data were normalized to a genomic region located within *GUSB* gene and represented as fold enrichment relative to the IgG control. Primer sequences used for qPCR amplification are described in Additional file 1.

### Tumor growth measurement

Congenitally athymic female nude mice (NCr-nu/nu) were purchased from the National Cancer Institute. Mice were treated as per the guidelines of our institutional animal care and use committee (IACUC). S2-013 cells (5 × 10^5^) were used for orthotopic injections into the pancreas of nude mice. After 7 days of implantation, mice were divided in groups of nine animals each and fed *ad libitum* with a normal diet or a ketogenic diet (composition given in Table S2 in Additional file 1). After 3 weeks of treatment, mice were sacrificed and tumor weight, tumor volume, muscle weight, carcass weight, etc. were recorded. Tumor tissue and other organs were flash frozen in liquid nitrogen for further analysis. Animal protocols were in accordance with the NIH Guide for the Care and Use of Laboratory Animals and were approved by the University of Nebraska Medical Center Animal Care and Use Committee.

### Immunohistochemistry

Immunohistochemistry was performed as described previously [26]. Ki67 (Thermo Fisher Scientific, Waltham, MA, USA), c-Myc (Epitomics, Burlingame, CA, USA), and Cleaved Caspase 3 (Cell Signaling Technology) primary antibodies were utilized. The stained sections were imaged at × 20 under an upright microscope and representative images were captured and presented.

### Metabolite extraction and NMR sample preparation

After confirming the confluence of the cells, the media was aspirated and the cells were washed twice with 1× phosphate buffer to remove remnants of the media before lysing the cells. The cells were then cold shocked with 1 mL of cryogenically cold 80% methanol/water mixture. The plates with the 80% methanol/water were incubated in a −80°C freezer for at least 15 min. The cells from the cold plates were scraped with a cell scraper and pipetted into an Eppendorf tube and centrifuged at 13,000 rpm for 5 min. The supernatant was collected and 250 μL of Milli-Q water (Millipore, Billerica, MA, USA) was added to the remaining cell debris for re-extraction. After mixing the cell debris with the water by pipetting, the sample was again centrifuged at 13,000 rpm for 5 min. The new supernatant was combined with the previously collected supernatant. Finally, the sample was dried using speed vacuum evaporator (SpeedVac® Plus, Savant, Thermo Scientific, Waltham, MA) to evaporate the methanol and subjected to freeze drying (Labconco, Kansas City, MO) to lyophilize the water consecutively. The dried sample was made ready for an NMR experiment by dissolving in 600 μL of 50 mM phosphate buffer in 99.8% D_2_O (Isotec, St. Louis, MO) at pH 7.2 (uncorrected) with 50 μM 3-(tetramethysilane) propionic acid-2,2,3,3-d_4_ (TMSP) (500 μM for 2D ^1^H-^13^C HSQC) for spectral referencing.

### NMR experiment and data analysis

The NMR spectra were acquired on a Bruker AVANCE DRX 500 MHz spectrometer equipped with 5 mm triple-resonance cryogenic probe (^1^H, ^13^C, and ^15^ N) with a Z-axis gradient. The NMR data collection was automated using a BACS-120 sample changer, ATM (automatic tuning and matching), and Bruker IconNMR™ software. The one-dimensional (1D) proton nuclear magnetic resonance (^1^H NMR) data was acquired using an excitation sculpting pulse sequence to remove the solvent peak and maintain a flat baseline [27]. The spectra were collected at 300 K with 32 K data points, 128 scans, 16 dummy scans, and a spectral width of 5,483 Hz. Our MVAPACK software (http://bionmr.unl.edu/mvapack.php) [28] was used to process the 1D ^1^H NMR spectra. The raw NMR data was only Fourier transformed and automatically phased. The resulting NMR spectrum was binned using an adaptive intelligent binning algorithm that automatically adjusts bin sizes to avoid splitting NMR resonances between multiple bins [29]. The spectral region before the TMSP was used as a training set to remove the noise from the data using the method stated by Halouska *et al.*[30]. The spectra were then normalized using standard normal variate (SNV) and scaled using Pareto scaling. The processed data was utilized to generate plots of principal component analysis (PCA) and orthogonal projections to latent structures discriminant analysis (OPLS-DA) scores and backscaled loadings (Additional file 2) using our MVAPACK software [28]. Metabolite identification from the 1D ^1^H NMR spectra was accomplished using the Chenomx NMR Suite 7.6 (http://www.chenomx.com/) and the backscaled loadings.

The 2D ^1^H-^13^C hetero-nuclear single quantum coherence (HSQC) NMR spectra were collected at 300 K with 64 scans, 16 dummy scans, and a 1.5-s relaxation delay. The spectra were collected with 2 K data points and a spectrum width of 4,735 Hz in the direct dimension and 64 data points and a spectrum width of 17,607 Hz in the indirect dimension. The 2D ^1^H-^13^C HSQC NMR spectra were processed using NMRPipe (NIH, Bethesda, Maryland) [31] and analyzed using NMRViewJ Version 8.0.3. Peak intensities were normalized by the average peak intensity for a given spectrum and then assigned to a metabolite using chemical shift references from the Human Metabolomics Database [32], Madison Metabolomics Consortium Database [33], and Platform for RIKEN Metabolomics [34]. Chemical shift errors of 0.08 and 0.25 ppm for the ^1^H and ^13^C chemical shifts, respectively, were used to match the experimental chemical shifts with the databases. In addition to chemical shifts, peak splitting patterns and peak shapes were also used to verify metabolite assignments.

The 2D ^1^H-^13^C HSQC NMR experiment is a more reliable approach for metabolite identification because of the significantly higher signal dispersion, and the correlation between ^1^H and ^13^C chemical shifts for each C-H pair in a molecule [35]. More importantly, the 2D ^1^H-^13^C HSQC experiment simplifies the analysis of the metabolome because only compounds containing a ^13^C-carbon derived from the ^13^C_6_-glucose added to the media will be detected. Thus, using ^13^C_6_-glucose will highlight metabolite changes associated with the glycolytic flux in tumor cells. This avoids the challenge with the 1D ^1^H NMR experiments where the spectra were dominated by catabolic products of ketone bodies. Thus, the identification of metabolites from the 2D ^1^H-^13^C HSQC experiments is more reliable and pertinent to the analysis of metabolic changes resulting from ketone body effects on pancreatic cancer cachexia.

Each metabolite peak from the two sets (control and ketone body-treated) of triplicate 2D ^1^H-^13^C HSQC NMR spectra were further normalized by using the maximum peak intensity for the metabolite and then scaled from 0 to 100. The peak intensities for each metabolite were then averaged and compared between the control and treated groups using a Student’s *t* test. Metabolites with a *p* value <0.1 were used to generate a heat map using the R statistical package [36]. A relative change in peak intensity implies a corresponding metabolite concentration change. Absolute concentrations are not measurable from the 2D ^1^H-^13^C HSQC because other factors, such as coupling constants, relaxation, and dynamics, also contribute to peak intensities.

### Measurement of blood glucose and β-hydroxybutyrate concentration

Blood glucose level of mice was measured after 16 h of starvation by utilizing Contour USB blood glucose meter (Bayer Health Care, Mishawaka, Japan), as per the manufacturer’s protocol. Blood ketone level was measured by utilizing a blood glucose and ketone monitor (Nova Biomedical, Waltham, MA, USA) as per the manufacturer’s protocol. The concentration of β-hydroxybutyrate was measured by comparing the TMSP-normalized methyl peaks of ^1^H NMR collected for six animals.

### C2C12 and 3T3L1 differentiation and conditioned medium preparation

C2C12 mouse myoblasts were grown in DMEM with 10% FBS. To induce differentiation, cells were switched to 2% horse serum and 10 μg/mL insulin-containing DMEM and grown for 72 h. 3T3L1 mouse embryo fibroblasts were cultured in DMEM with 10% FBS. For differentiation of 3T3L1 preadipocytes, after 2 days at confluency, cells were treated with differentiation medium: DMEM containing 10% FBS, 1 μM dexamethasone, 0.5 mM methylisobutylxanthine (IBMX), and 1 μg/mL insulin for 2 days. After 2 days, differentiation medium was replaced with DMEM containing 10% FBS. Medium was changed regularly after 48 h. For conditioned medium preparation, cells were plated at a density of 50,000 cells/cm^2^, and after 12 h of seeding, cells were washed twice with PBS and cultured in serum-free DMEM for the next 24 h. Conditioned medium was centrifuged at 1,200 *g* for 10 min and filtered with a 0.2-μm syringe filter and used immediately or stored at −80°C.

### Measurement of intracellular pH

Cytosolic pH was measured by using fluorescence spectroscopy using BCPCF-AM as described by Marino *et al.*[37].

### Statistical analysis

Comparisons between different groups were performed by using ANOVA (one-way; GraphPad Prism version 4.03) with Dunnett’s post hoc test. Student’s *t* test was used for *in vivo* studies. A *p* value of <0.05 was considered to be significant.

## Results

### Ketone bodies diminish pancreatic cancer cell growth and induce apoptosis in a dose-dependent manner

**Figure 1:**
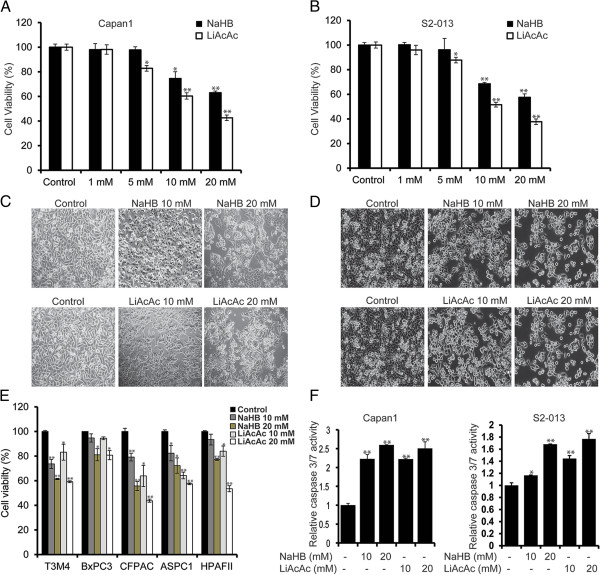
**Ketone bodies inhibit growth and induce apoptosis in pancreatic cancer cell lines.** Capan1 **(A)** and S2-013 **(B)** cells were treated with different concentrations of sodium-3-hydroxybutyrate (NaHB) and lithium acetoacetate (LiAcAc) for 72 h, and cell viability was determined by MTT assay. *Bar* represents percent viability under indicated treatments relative to treatment with solvent control. Representative bright-field images of Capan1 **(C)** and S2-013 **(D)** cells under treatment with 10- and 20-mM concentrations of NaHB and LiAcAc for 72 h. **(E)** Multiple pancreatic cancer cell lines were treated with 10- and 20-mM concentrations of NaHB and LiAcAc for 72 h, and relative cell viability determined by MTT assay is plotted in the *bar charts*. **(F)** Capan1 and S2-013 cells treated with 10- and 20-mM concentrations of sodium-3-hydroxybutyrate and lithium acetoacetate for 48 h and the relative caspase 3/7 activity are plotted. Values represented are mean ± SEM. *P < 0.05; **P < 0.01.

We investigated the effect of ketone bodies (sodium hydroxybutyrate and lithium acetoacetate) on cell survival in multiple pancreatic cancer cell lines. Initially, we evaluated the effect of multiple doses of sodium hydroxybutyrate and lithium acetoacetate (1 to 20 mM) on the survival of Capan1 and S2-013 pancreatic cancer cells by performing MTT assays. Ketone bodies were observed to inhibit cell survival in a dose-dependent manner. Ketone bodies significantly inhibited cell growth at concentrations of 10 and 20 mM after a 72-h treatment (Figure 1A,B). To evaluate if the ketone body-induced survival diminution is specific to cancer cells, we subjected immortalized, non-transformed pancreatic epithelial cell lines HPNE and RAPAN to incubation with sodium hydroxybutyrate and lithium acetoacetate. We observed no significant effect on survival of these cells under treatment with ketone bodies (Additional file 3). Similarly, to rule out the possibility of organic acid and lithium ion being responsible for reduction in cancer cell survival, rather than ketone bodies themselves, we evaluated the survival of S2-013 and Capan1 under treatment with *S*-hydroxy butyric acid and lithium chloride. We observed no significant effect on S2-013 and Capan1 cell survival after 72 h of treatment (Additional file 4). Furthermore, we investigated the effect of sodium hydroxybutyrate, lithium acetoacetate, and *S*-hydroxybutyrate on intracellular pH. After 6 h of treatment with different doses of sodium hydroxybutyrate, lithium acetoacetate, and *S*-hydroxybutyrate on S2-013, Capan1, HPNE, and RAPAN cells, we observed significant decrease in intracellular pH (0.2 to 0.4 units) in all the cell lines (Additional file 5). Although intracellular acidification upon treatment with ketone bodies might contribute to the antiproliferative effects of ketone bodies, it is not the primary cause of cells death as we did not observe significant cell death upon treatment with *S*-isomer of hydroxybutyrate that caused similar intracellular pH change as did the other ketone bodies. Furthermore, we observed a similar effect on intracellular pH in non-transformed pancreatic epithelial cell lines HPNE and RAPAN after treatment with sodium hydroxybutyrate, lithium acetoacetate, and *S*-isomer of hydroxybutyrate but no significant cell death, as mentioned earlier. Bright-field microscopy images of the cells treated with 10 and 20 mM of NaHB and LiAcAc are presented in Figure 1C,D. Furthermore, we investigated the effect of ketone bodies on five pancreatic cancer cell lines and observed that cell growth is significantly inhibited in a dose-dependent fashion (Figure 1E). We also investigated the effect of ketone body treatment on caspase activity in Capan1 and S2-013 cell lines. Caspase 3/7 activity increased upon treatment of the pancreatic cancer cells with ketone bodies in a dose-dependent manner (Figure 1F).

### Ketone bodies cause metabolic alterations in pancreatic cancer cells

**Figure 2:**
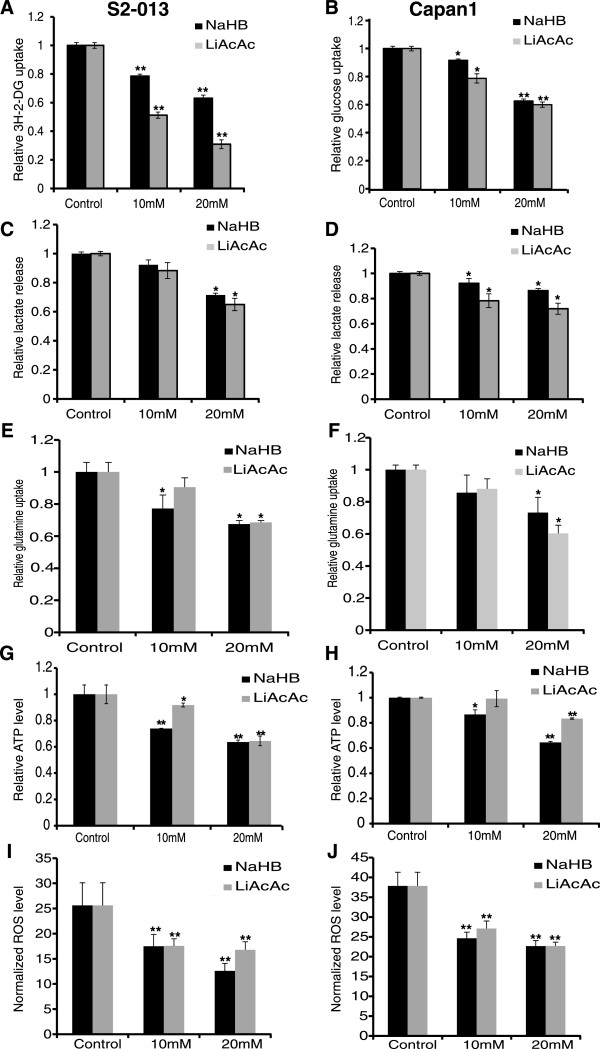
**Ketone bodies induce metabolic alterations in pancreatic cancer cell lines.** S2-013 **(A)** and Capan1 **(B)** cells were treated with different doses of ketone bodies for 24 h, and glucose uptake was determined by performing ^3^H-2DG uptake assay. *Bars* represent counts normalized with cell number and plotted relative to control. Lactate release was determined by colorimetric assay using culture medium of S2-013 **(C)** and Capan1 **(D)** cells treated with different concentrations of NaHB and LiAcAc for 24 h. Values were normalized with total cell number and represented relative to controls. S2-013 **(E)** and Capan1 **(F)** cells were treated with indicated concentrations of ketone bodies for 24 h, and glutamine uptake was determined by performing tritiated Glutamine, l-[3,4-^3^H(N)] uptake assays. Counts were normalized with cell number and plotted relative to control. ATP levels in S2-013 **(G)** and Capan1 **(H)** cells post 24-h treatment with ketone bodies were determined by performing ATP bioluminescence assays. Values were normalized to total protein concentration and represented relative to control. Reactive oxygen species level of S2-013 **(I)** and Capan1 **(J)** cells under treatment with ketone bodies was determined by utilizing a fluorescence probe, dihydroethidium (DHE), and fluorescence intensity normalized to cell count was plotted. Values represented are mean ± SEM. *P < 0.05; **P < 0.01.

Previous studies indicate that a ketogenic diet induces metabolic alterations in mice [17]. Hence, we next investigated the effect of ketone bodies on pancreatic cancer cell metabolism. Because cancer cells demonstrate an increase in glycolysis [38], we examined the effect of ketone bodies on glucose uptake and lactate release in Capan1 and S2-013 cells. Treatment of Capan1 and S2-013 cells with ketone bodies resulted in a decrease in glucose uptake (Figure 2A,B) and release of lactate (Figure 2C,D) in a dose-dependent manner. Since glutamine also supports pancreatic cancer cell growth [7], we also evaluated the effect of ketone bodies on glutamine uptake. Our results indicate a reduced uptake of glutamine by Capan1 and S2-013 pancreatic cancer cells under treatment with ketone bodies (Figure 2E,F). Furthermore, we observed a reduction in intracellular ATP levels upon treatment with ketone bodies (Figure 2G,H). Reactive oxygen species (ROS; a natural by-product of oxidative metabolism) levels were also downregulated upon treatment with ketone bodies (Figure 2I,J). Overall, our results indicate that ketone bodies diminish the overall energetic health of pancreatic cancer cells by reducing glucose uptake, lactate release, glutamine uptake, cellular ATP content, and ROS levels.

### Ketone bodies diminish the expression of glycolytic enzymes

**Figure 3:**
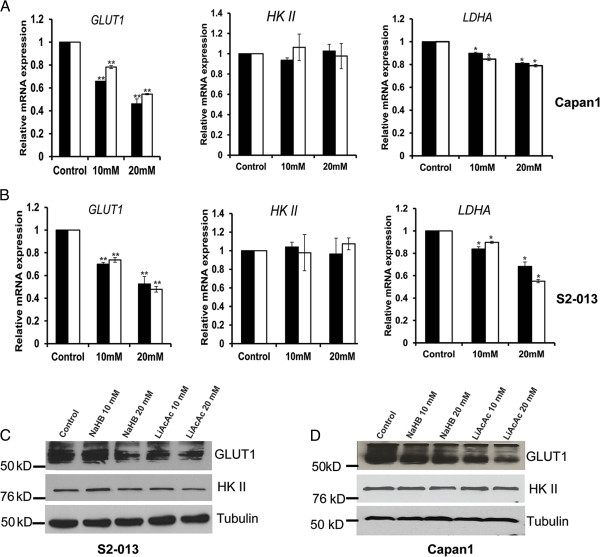
**Ketone bodies repress the expression of key glycolytic enzymes.** Relative mRNA expression levels of *GLUT1*, *HKII*, and *LDHA* in Capan1 **(A)** and S2-013 **(B)** cells treated with 10- and 20-mM concentrations of NaHB and LiAcAc for 24 h. Total RNA was isolated from NaHB- and LiAcAc-treated as well as control cells, and relative mRNA levels of different genes were determined by performing qRT-PCR. β-Actin was utilized as an internal control. Protein expression of GLUT1 and HKII was determined by immunoblotting the total cell lysates from S2-013 **(C)** and Capan1 **(D)** cells treated with 10 and 20 mM NaHB and LiAcAc for 48 h. β-Tubulin was utilized as an internal control. Values shown are mean ± SEM. *P < 0.05; **P < 0.01.

To determine the mechanism of reduced glucose uptake and lactate release upon treatment of pancreatic cancer cells with ketone bodies, we next examined if ketone body-mediated changes were due to alterations in gene expression levels. We evaluated the expression of genes encoding glucose transporter-1 (*GLUT1*) and glycolytic enzymes hexokinase II (*HKII*) and lactate dehydrogenase A (*LDHA*) in ketone body-treated and control cells. In both Capan1 and S2-013 cells, we observed a reduced expression of *GLUT1* and *LDHA* but no significant change in *HKII* expression (Figure 3A,B) upon treatment with ketone bodies. Furthermore, we analyzed the protein expression levels of GLUT1 and HKII in S2-013 and Capan1 cells under treatment with ketone bodies or control. Our results indicate a reduced expression of GLUT1 but almost no change in HKII levels in cells treated with ketone bodies (Figure 3C,D). To eliminate the possibility of non-specific effects of organic acid and lithium ion on gene expression and metabolism, we determined GLUT1, HKII, and LDHA gene expression and glucose uptake in S2-013 cells after treatment with *S*-hydroxy butyric acid and lithium chloride. We found a negligible effect of these agents on metabolic gene expression and glucose uptake (Additional file 6).

### Ketone bodies diminish c-Myc expression and activity

**Figure 4:**
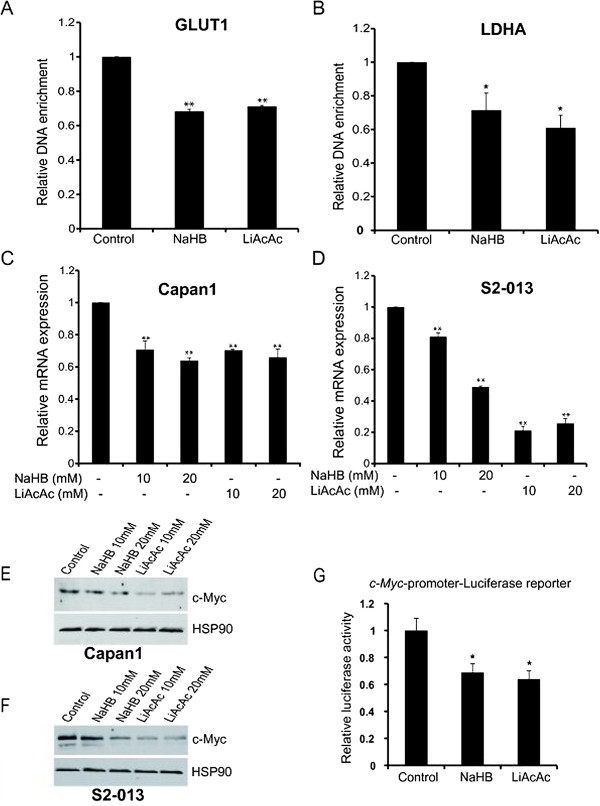
**Ketone bodies reduce c-Myc expression and its recruitment to glycolytic gene promoters.** Recruitment of c-Myc onto *GLUT1***(A)** and *LDHA***(B)** promoters in S2-013 cells under treatment with 20 mM NaHB, LiAcAc, or control was confirmed by performing ChIP using anti-c-Myc Ab and IgG control, followed by qRT-PCR analysis. Relative c-Myc mRNA levels in Capan1 **(C)** and S2-013 **(D)** cells treated with 10 and 20 mM NaHB, LiAcAc, or control for 24 h. Total RNA was isolated and relative mRNA level of *c-Myc* was determined by qRT-PCR. β-Actin was utilized as an internal control. Capan1 **(E)** and S2-013 **(F)** cells were treated with indicated doses of ketone bodies for 48 h, and c-Myc protein level was determined by immunoblotting the whole cell lysates. HSP90 was used as an internal control. **(G)** c-Myc-promoter-firefly luciferase reporter and *Renilla* luciferase reporter plasmids were transiently transfected into S2-013 cells. After 16 h of transfection, cells were treated with solvent control or ketone bodies for 24 h. Normalized firefly to *Renilla* luciferase activity ratio is plotted in the *bar chart*. Values represented are mean ± SEM. *P < 0.05; **P < 0.01.

Since we observed a downregulation of glucose and glutamine uptake upon treatment with ketone bodies and a corresponding decrease in glycolytic gene expression, we next investigated the molecular mechanism behind this decrease. Specifically, we evaluated the effect of ketone bodies on the activity and expression of c-Myc, a common regulator of both glucose and glutamine metabolism [39]. Using the TFsearch program, the promoter sequences of *GLUT1* and *LDHA* were both found to contain c-Myc binding sites. Chromatin immunoprecipitation assays were then performed to evaluate the c-Myc occupancy at the *GLUT1* and *LDHA* promoters. Our results indicate a reduced occupancy of c-Myc on *GLUT1* and *LDHA* promoters for S2-013 cells treated with ketone bodies (Figure 4A,B). Furthermore, we performed real-time PCR to determine the *c-Myc* transcript levels in S2-013 and Capan1 cells after treatment with ketone bodies or control. We observed significant reduction in *c-Myc* expression after treatment with ketone bodies (Figure 4C,D). We also evaluated c-Myc protein expression after treatment with ketone bodies and observed a reduction in c-Myc levels in a dose-dependent manner (Figure 4E,F). To confirm if the alterations in c-Myc expression were due to altered c-Myc transcription, we evaluated the transcriptional activity of c-Myc promoter by utilizing c-Myc promoter-luciferase reporter constructs [24]. We observed significantly reduced c-Myc promoter activity under treatment with ketone bodies (Figure 4G). Altogether, our results demonstrate that the treatment of pancreatic cells with ketone bodies leads to a reduced *c-Myc* expression at mRNA level and a reduced c-Myc occupancy on the *GLUT1* and *LDHA* promoters.

### Ketone bodies inhibit tumor cell-induced muscle fiber and adipocyte degradation in cell-based assays

**Figure 5:**
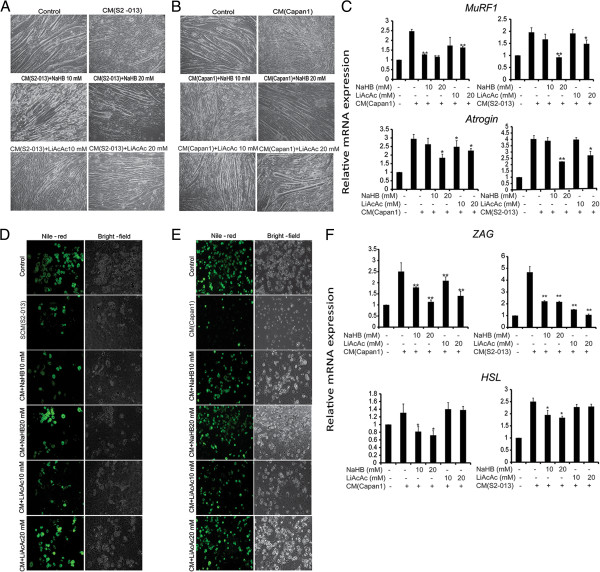
**Ketone bodies inhibit tumor cell-conditioned medium-induced degradation of myofibers and adipolysis.** Differentiated C2C12 cells were treated with S2-013 **(A)** and Capan1 **(B)** cell-conditioned medium with or without solvent control and 10 and 20 mM NaHB and LiAcAc for 72 h, and bright-field images were represented for individual treatments. **(C)** Differentiated C2C12 cells were cultured in Capan1 and S2-013 cell-conditioned medium with or without ketone body treatment for 24 h. Total RNA was isolated and relative mRNA levels of *MuRF1* and *Atrogin* were determined by performing qRT-PCR. β-Actin was utilized as an internal control. Differentiated 3T3L1 cells were cultured in S2-013 **(D)** and Capan1 **(E)** cell-conditioned medium with or without ketone body treatment for 72 h and stained with nile red. Fluorescent and bright-field images for individual treatments are presented. **(F)** Differentiated 3T3L1 cells were cultured in Capan1 and S2-013 cell-conditioned medium with or without ketone body treatment for 24 h. Total RNA was isolated and relative mRNA levels of *Zag* and *HSL* were determined by qRT-PCR. β-Actin was utilized as an internal control. Values represented are mean ± SEM. All statistical analyses were conducted with one-way ANOVA with Dunnett’s post hoc test and CM as the reference group. *P < 0.05; **P < 0.01.

Malignant cells have been shown to lack key mitochondrial enzymes required for metabolizing ketone bodies to produce ATP, while muscle cells retain this capacity [18]. Of note, β-hydroxybutyrate improves body weight, while reducing proteolysis in muscle cells [40]. Hence, we developed a cell culture-based system to evaluate the effect of ketone bodies on muscle fibers and adipocyte adipose deposits under coculture with cancer cells. This was achieved by differentiating C2C12 premyocytes into myotubes and 3T3L1 preadipocytes into differentiated adipocytes. Treatment with Capan1 or S2-013 cancer cell-conditioned medium (CCCM) induced degradation of myotubes and depletion of adipose deposits in 3T3L1 adipocytes. Treatment with ketone bodies demonstrated a significant protection of myotubes (Figure 5A,B) and adipocytes (Figure 5D,E) against CCCM. Furthermore, we analyzed the gene expression levels of *muscle-specific ring finger protein 1* (*MuRF1* or *Trim63*) and *Atrogin* (*Fbxo32*; also known as *muscle atrophy F-box protein* or *MAFbx*) in myotubes, and *zinc alpha-2-glycoprotein 1* (*Zag* or *Azgp1*) and hormone-sensitive lipase (*HSL* or *Lipe*) in adipocytes treated with CCCM under treatment with ketone bodies or control. These proteins are upregulated during cancer-induced cachexia [41]. Ketone bodies were shown to significantly lower *MuRF1* and *Atrogin* mRNA levels even in the presence of CCCM (Figure 5C). We also observed a significant decrease in *Zag* and *HSL* expression upon treatment with ketone bodies (Figure 5F). Hence, our results indicate the utility of ketone bodies in inhibiting cancer-induced cachexia.

### Ketone bodies alter central carbon metabolism in pancreatic cancer cells

**Figure 6:**
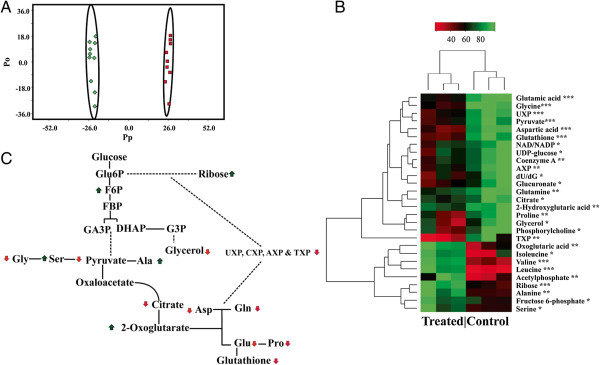
**Ketone bodies modulate metabolite levels in pancreatic cancer cells. (A)** OPLS-DA score plot generated from 1D ^1^H NMR spectra collected from cell lysates of S2-013 cells (*red square*) and S2-013 cells treated with 20 mM NaHB (*green diamond*); each *point* in the OPLS-DA score plot represents a single 1D ^1^H NMR spectrum. *Ellipses* enclose the 95% confidence intervals estimated by the sample means and covariances of each class. The leave-n-out cross validation yielded a quality assessment (*Q*^2^) value of 0.959 and *R*^2^ value of 0.997. The OPLS-DA model was validated using CV-ANOVA yielding a *p* value of 8.74 × 10^−6^. **(B)** Heat map generated from 2D ^1^H-^13^C HSQC NMR spectral data for S2-013 cells. The *heat map* represents triplicate measurements of metabolite intensities recorded (*P < 0.1; **P < 0.05; ***P < 0.001). **(C)** Metabolic pathway depicts ^13^C carbon flow from glucose to the intermediates of glycolytic pathway, citric acid cycle, amino acid metabolism, and nucleotide analogues. The *arrows* represent relative increase (*green arrow up*) or decrease (*red arrow down*) in metabolite concentrations due to ketone body treatment.

To evaluate the effect of ketone bodies on metabolite levels in pancreatic adenocarcinoma cells, we performed a series of NMR-based metabolomics studies. S2-013 cells were treated with 20 mM sodium-3-hydroxybutyrate for 24 h, and then a set of 1D ^1^H NMR spectra were acquired for cell extracts of sodium-3-hydroxybutyrate-treated or control S2-013 cells. OPLS-DA of the NMR spectra indicates that the metabolomes from the ketone body-treated cells and the control cells clustered into two separate groups in a OPLS-DA score plot, indicating that the treated cancer cells are metabolically differentiated from the controls (Figure 6A). The OPLS-DA model was validated using CV-ANOVA yielding a *p* value of 8.74 × 10^−6^ (Additional file 2). The backscaled loadings (Additional file 7) of the 1D ^1^H NMR data indicate a reduction in cellular glutamine and glutamate level. Many cancers are dependent on glutamine metabolism and have enhanced glutamine uptake. A significant reduction of choline-containing compounds is also observed in ketone body-treated cells. Total choline-containing compound concentration is typically increased in multiple cancer phenotypes and referred as a metabolic hallmark of cancer. It is particularly noteworthy that ketone body treatment appears to reverse these trends [42]. Ketone body treatment also elevates myo-inositol, taurine, and alanine. Myo-inositol and its derivative are known to suppress pancreatic cancer [43].

To further explore the impact of ketone bodies on other metabolic process, we then labeled the S2-013 metabolome with ^13^C_6_-glucose and compared the cell extracts from sodium-3-hydroxybutyrate-treated S2-013 cells with control cells using 2D ^1^H-^13^C HSQC NMR experiments. The 2D ^1^H-^13^C HSQC spectra identified the ^13^C-labeled metabolites in the cell extracts derived from ^13^C_6_-glucose. Importantly, ^13^C_6_-glucose highlighted metabolite changes associated with glycolytic flux in tumor cells. The NMR metabolomics studies identified multiple metabolites exhibiting statistically significant concentration changes due to ketone body treatment (Figure 6B). These identified metabolites were then incorporated into a network using Cytoscape [44] and Metscape [45] by linking nearest-neighbor metabolites. Overall, glucose-derived metabolites involved in glycolysis, amino acid metabolism, and TCA cycle were altered upon treatment with ketone bodies (Figure 6C). The 1D ^1^H loadings are also consistent with this general observation. Specifically, both experiments identify changes in amino acid metabolism. Again, the ketone bodies are being used as a metabolic substrate and are replacing the cellular need for glucose and glycolysis. The importance of a detailed analysis of metabolic changes is highlighted by a key example. The 2D ^1^H-^13^C HSQC experiments indicate that the concentrations of glutamate and glutamine derived from glucose have decreased significantly. Similarly, the 1D ^1^H loadings indicate that the *overall* concentrations of glutamate and glutamine have also decreased upon ketone body treatment. The metabolites derived from ketone bodies do not contain a ^13^C-carbon and are not detected in the 2D ^1^H-^13^C HSQC experiment. Correspondingly, the 1D ^1^H loadings identify major changes for unlabeled metabolites that are not visible in a 2D ^1^H-^13^C HSQC spectrum. Thus, the 2D ^1^H-^13^C HSQC experiments are complimentary to the 1D ^1^H experiments and provide a more detailed analysis of a specific subset of the metabolome.

To study if ketone bodies are getting metabolized by cancer cells, we treated S2-013 cells with ^13^C_4_-labeled hydroxyl butyrate and identified its catabolic products by using a 2D ^1^H-^13^C HSQC NMR experiment (Additional file 8). Because of the low natural abundance of ^13^C (1.1%), the only peaks observable in a 2D ^1^H-^13^C HSQC spectrum must originate from the ^13^C_4_-labeled hydroxyl butyrate. As a reference point, the cellular extract was spiked with 500 μM of TMSP. The single peak originating from the natural abundant TMSP ^13^C-methyl groups is barely detectable in the 2D ^1^H-^13^C HSQC spectrum. A negative control (*data not shown*), where S2-013 cells are not treated with a ^13^C-labeled metabolite, yielded a null spectrum. Thus, the fact that multiple intense peaks are observable in the 2D ^1^H-^13^C HSQC spectrum is a clear evidence that the S2-013 cells uptake hydroxyl butyrate. The ^1^H and ^13^C chemical shifts measured from the 2D ^1^H-^13^C HSQC spectrum were then compared against reference NMR spectra available from the Human Metabolomics Database [32], Madison Metabolomics Consortium Database [33], and Platform for RIKEN Metabolomics [34] to identify other ^13^C-labeled metabolites present in the cell extract. We observed chemical shifts consistent with 3-hydroxybutyrate, the original ^13^C-carbon source, and betaine, *N*-acetylglucosamine, homocarnosine, and succinate, which all must be catabolic products of 3-hydroxybutyrate.

### Inhibiting glycolytic flux in tumor cells prevents cachexia phenotype in cell line models

**Figure 7:**
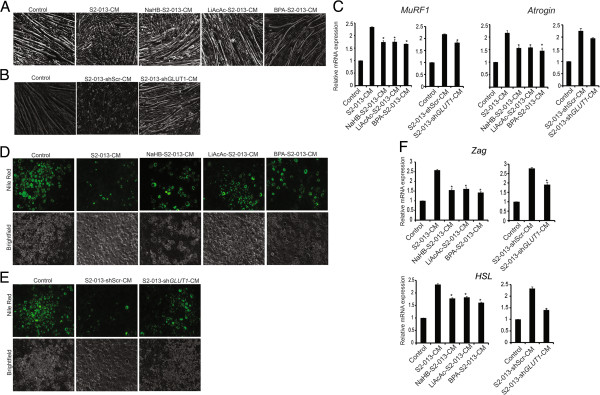
**Pretreatment of tumor cells with ketone bodies or glycolytic inhibition diminishes their cachectic potential.** S2-013 cells were treated with solvent control, 20 mM NaHB (NaHB-S2-013), 20 mM LiAcAc (LiAcAc-S2-013), and 10 μM 3-bromopyruvic acid (BPA-S2-013) for 24 h. The cells were then washed twice with phosphate-buffered saline and cultured in serum-free DMEM. After 24 h, the conditioned medium was collected. The conditioned medium was also prepared from GLUT1 knockdown S2-013 (S2-013-sh*GLUT1*) and control cells (S2-013-shScr). Differentiated myotubes from C2C12 cells were cultured in **(A)** control, S2-013-CM, NaHB-S2-013-CM, LiAcAc-S2-013-CM, and BPA-S2-013-CM or **(B)** control, S2-013-shScr-CM, and S2-013-sh*GLUT1*-CM for 72 h, and bright-field images were represented for individual treatments. Differentiated 3T3L1 cells were cultured in **(C)** control, S2-013-CM, NaHB-S2-013-CM, LiAcAc-S2-013-CM, and BPA-S2-013-CM or **(D)** control, S2-013-shScr-CM, and S2-013-sh*GLUT1*-CM for 72 h and stained with nile red, and images for individual treatments are represented. **(E)** Differentiated myotube form C2C12 cells were cultured in similar conditions for 24 h. Total RNA was isolated and relative mRNA levels of *MuRF1* and *Atrogin* were determined by qRT-PCR. β-Actin was utilized as an internal control. **(F)** Differentiated 3T3L1 cells were cultured in the above-mentioned conditions for 24 h. Total RNA was isolated and relative mRNA levels of *Zag* and *HSL* were determined by qRT-PCR. β-Actin was utilized as an internal control. Values represented are mean ± SEM. All statistical analyses were conducted with one-way ANOVA with Dunnett’s post hoc test and S2-013-CM as the reference group.*P < 0.05; **P < 0.01.

Metabolic alterations in cancer cells [46] may contribute to the secretion of cachectic cytokines and metabolites involved in cancer-induced cachexia. Based on our findings that ketone bodies mediated a decrease in the expression of *GLUT1* resulting in a metabolic flux, we evaluated if inhibiting the glycolytic flux alters the cachectic potential in cancer cells. We pretreated S2-013 cells with 3-sodium hydroxybutyrate, lithium acetoacetate, or glycolytic inhibitor 3-bromopyruvic acid (BPA) and then collected the conditioned medium and evaluated the cachectic potential by utilizing C2C12 myotube and 3T3L1 adipocyte systems. We also assayed conditioned medium from *GLUT1* knockdown S2-013 cells (S2-013-sh*GLUT1*) for cachectic potential. Treatment with conditioned medium generated from ketone bodies or glycolytic inhibitor-pretreated S2-013 cells, or GLUT1 knockdown S2-013 cells, demonstrated a significant protection against myotube degradation (Figure 7A,B) and adipocyte fat depletion (Figure 7C,D) in comparison to controls. Furthermore, we analyzed the gene expression levels of *MuRF1* and *Atrogin* in myotubes and *Zag* and *HSL* in adipocytes treated with the above-described CCCM. We observed a significant reduction of *MuRF1* and *Atrogin* gene expression in C2C12 myotubes under treatment with CCCM from ketone body or inhibitor-pretreated cells, or GLUT1 knockdown cell-derived conditioned media, in comparison to control CCCM-treated myotubes (Figure 7E). Similarly, we observed a reduced expression of *Zag* and *HSL* in 3T3L1 adipocytes in the presence of pretreated CCCM in comparison to the controls (Figure 7F). To ensure the inhibition of glycolysis by BPA and GLUT1 knockdown, we performed glucose uptake assay in S2-013 cells treated with BPA or solvent control as well as S2-013-sh*GLUT1* and S2-013-shScr cells. We observed a significant reduction in glucose uptake under these conditions (Additional file 9). As we observed that ketone bodies act like metabolic inhibitors, we further explored if glycolytic product lactate and alanine, which could be secreted into the medium, in CCCM could abrogate the effect of ketone body treatment of tumor cells on the expression of cachectic markers in adipocytes and myotubes. We observed no significant alteration in cachectic marker expression after adding lactate or alanine (Additional file 9), which indicates that these metabolic by-products are not solely responsible for the cachectic phenotype.

### A ketogenic diet reduces tumor growth and cachectic phenotype in animal models

**Figure 8:**
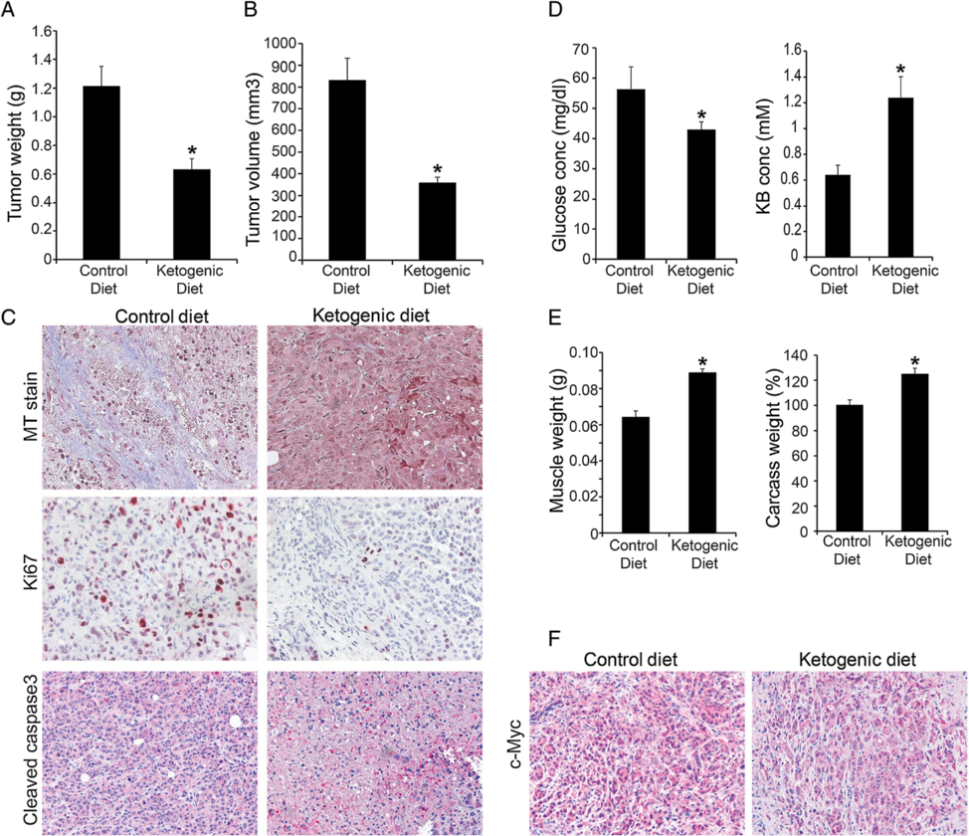
**A ketogenic diet reduces tumor growth and proliferation and reverts the cachectic phenotype.** Into the pancreas of athymic nude mice, 0.5 × 10^6^ S2-013 cells were orthotopically implanted. After 1 week of implantation, mice were divided in two groups and fed with either a control diet or a ketogenic diet. Three weeks post treatment, mice were sacrificed and tumor weight **(A)** and tumor volume **(B)** were measured. **(C)** Masson’s trichrome staining (blue stain indicates desmoplastic region) and immunohistochemistry images from the control diet and the ketogenic diet-fed mice tumor sections. **(D)** Blood glucose and ketone levels in the control and the ketogenic diet-fed mice before necropsy. **(E)** Muscle weight and carcass weight of the control and the ketogenic diet-fed mice. **(F)** Immunohistochemistry of c-Myc in the control and the ketogenic diet-fed mice tumor sections. Values shown are mean ± SEM. *P < 0.05; **P < 0.01.

To determine the effect of a ketogenic diet on tumor growth, we implanted S2-013 pancreatic cancer cells orthotopically into the pancreas of athymic nude mice. One week later, S2-013 cell-implanted mice were fed *ad libitum* on a ketogenic diet or normal chow for 3 weeks. After 3 weeks on the diets, mice were sacrificed and tumor weight, tumor volume, muscle weight, and carcass weight were recorded. S2-013 tumor-bearing mice fed on the ketogenic diet demonstrated reduced tumor weight and tumor volume (Figure 8A,B). The ketogenic diet also reduced desmoplasia as observed by Masson’s trichrome stain (Figure 8C). Furthermore, we investigated the effect of the ketogenic diet on tumor cell proliferation and apoptosis by immunohistochemically staining the tumor sections for Ki67 and cleaved caspase 3, respectively. We observed that the ketogenic diet reduced tumor cell proliferation and increased apoptosis as indicated by the decreased percentage of cells with Ki67-positive staining and increased staining for cleaved caspase 3, in comparison to the tumors from mice fed on a control chow (Figure 8C). Blood glucose levels were also lower in the ketogenic diet-fed mice, while the ketone body levels (β-hydroxybutyrate) were significantly higher in the same group (Figure 8D). Furthermore, we performed NMR analysis to determine the levels of ketone bodies in plasma and tumor tissues from control and ketogenic diet-fed mice. We observed an approximately twofold increase of ketone body (β-hydroxybutyrate) concentration in tumor tissue (range 0.85–7.84 mM) in comparison to plasma (range 0.47–3.13 mM), as shown in Additional file 10. The difference between tumoral ketone body levels in ketogenic diet-fed and normal diet-fed mice was also very striking (Additional file 11), as observed by NMR. Furthermore, we investigated muscle weight and carcass weight as markers of cancer-induced cachexia in the control or the ketogenic diet-fed tumor-bearing mice. We observed a 45% increase in the muscle weight and a 20% increase in the carcass weight in ketogenic diet-fed mice in comparison to the controls (Figure 8E). We also evaluated c-Myc expression in tumor sections from control or ketogenic diet-fed mice. As expected from our cell culture-based studies, we observed reduced tumoral c-Myc expression in the ketogenic diet-fed mice (Figure 8F). Overall, our results indicate that a ketogenic diet diminishes tumor growth and proliferation in mice and inhibits cancer-induced cachexia.

## Discussion

Metabolism plays a very important role in cellular function and cell survival. Altered cell metabolism is a hallmark of cancer [6]. Cellular proliferation is directly dependent on nutrient availability, and most mitogenic signals exert their influence on cell proliferation by regulating nutrient uptake and synthesis of DNA, RNA, protein, and lipids [46]. It has been shown that enhanced glucose uptake supports the production of intermediates required for biomass production in proliferating cancer cells. In addition, cancer cells also demonstrate altered glutamine uptake and glutaminolysis, which replenish intermediates of tricarboxylic acid cycle and play an important role in the biosynthetic processes [47]. Our present studies indicate that ketone bodies revert metabolic adaptations in pancreatic cancer cells to induce growth arrest and apoptosis. Our results indicate a reduction in glucose uptake, glycolytic flux, glutamine uptake, lactate secretion, and ATP content in pancreatic cancer cells after treatment with ketone bodies. The reversal of metabolic syndrome in cancer cells by ketone bodies might be related to levels of c-Myc. Furthermore, we demonstrate that metabolic reprogramming of tumor cells by ketone bodies is responsible for diminishing cancer cell-induced cachexia in cell line models and animal models of pancreatic cancer.

Oxidative stress plays an important role in cancer progression, which results from an imbalance between the production of reactive oxygen species (ROS) and the cellular antioxidant defenses. ROS deregulates the redox homeostasis and promotes several inflammatory pathways leading to tumor formation [48]. Since ketone bodies were shown to diminish cellular glucose and glutamine flux, we also investigated if ketone bodies would diminish cellular ROS levels. We observed a reduction in ROS levels after treatment with ketone bodies. Of note, the anticancerous property of several phytochemicals and dietary compounds is mediated by their antioxidant activity [48]. Recent studies indicate that β-hydroxybutyrate, the main ketone body found in the body, reduces oxidative stress [49] and, in turn, functions as a histone deacetylase inhibitor. Inhibition of histone deacetylase activity can suppress or prevent cancer growth as indicated by several studies with histone deacetylase inhibitors that are currently being evaluated for cancer prevention [50].

Most tumors are highly dependent on glucose as an energy source. For the same reason, several inhibitors of glycolysis have been extensively evaluated in preclinical cancer models [51]. We observed a reduced expression of glucose transporter GLUT1 and glycolytic enzyme LDHA in pancreatic cancer cells upon treatment with ketone bodies (Figure 3). Inhibition of GLUT1, which is the main transporter of glucose in cancer cells, is currently being considered for cancer therapy [52]. Also, LDHA inhibition causes reduced cancer cell proliferation [47]. Furthermore, this present study indicates that ketone bodies diminish c-Myc expression and its occupancy on glycolytic gene promoters. c-Myc is an important regulator of cell growth and proliferation [39]. In transformed cells, c-Myc enhances the expression of glycolytic genes *GLUT1*, *LDHA*, and *ENO1* as well as glutaminolytic genes such as *GLS*[53,54]. c-Myc is considered an attractive therapeutic target due to its role in modulating cell metabolism, tumor initiation, and growth in a variety of cancer types [53]. Hence, reduced c-Myc expression by ketone bodies might contribute to the growth inhibitory effects of ketone bodies in pancreatic cancer. Previously, it has been reported that c-Myc inhibition leads to regression of lung cancer [55], bladder cancer [56], and pancreatic cancer [57].

Cachexia affects a majority of pancreatic cancer patients and significantly contributes to morbidity and mortality [3]. However, agents targeting cachexia remain largely elusive. Metabolic adaptations in tumor cells are primarily responsible for the muscle weight loss and the depletion of adipose deposits associated with cachexia. Our study identifies ketone bodies as therapeutic agents that can diminish cancer cachexia by deactivating metabolic adaptations in cancer cells. Herein, we described cell culture-based models for investigating agents targeting cancer cachexia that is a corollary to our animal models of pancreatic cancer cachexia. Our results with the cell culture-based models indicate that ketone bodies significantly inhibit myotube degradation and adipolysis. We also observed a reduced expression of *MuRF1*, *Atrogin*, *Zag*, and *HSL*, which are signature genes associated with cachexia (Figure 5). Thus, ketone bodies inhibit pancreatic cancer cell growth along with inhibition of pancreatic cancer-associated cachexia. Our NMR-based metabolomics studies indicate that treatments with ketone bodies significantly alter the metabolite flux in pancreatic cancer cells. We observed reduced cellular levels of glutamine, which is the most abundant amino acid and is involved in the regulation of growth and proliferation of cells. Cancer cell proliferation significantly depends on glutamine availability [13], and hence, the growth inhibitory effects of ketone bodies might in part be mediated by glutamine availability. Increased proline and lysine levels might play a role in growth inhibition of pancreatic cancer cells. Growth inhibitory effects of proline and lysine are known in bladder cancer cells [58]. We also observed an accumulation of the TCA metabolite citrate, which inhibits the phosphofructokinase enzyme in the glycolysis pathway and induces apoptosis in cancer cells [57]. Altogether, the metabolic shift after treatment of ketone bodies might significantly contribute to growth inhibition and apoptosis in cancer cells.

Although a link has been suggested between the metabolic needs of the growing tumor and cachexia syndrome, no studies have evaluated such a relationship. Our studies, for the first time, have confirmed that the metabolic requirements of growing tumor cells are directly responsible for the muscle degradation and lipolysis that are fundamental to the cachexia syndrome. Our results indicate that the inhibition of either glucose uptake by *GLUT1* knockdown or chemical inhibition of glycolysis by utilizing BPA in cancer cells diminished myotube degradation and depletion of adipose deposits. Hence, our findings underscore the significance of tumor cell glucose metabolism in facilitating tumor-associated cachexia.

We observed increased concentration of ketone bodies in tumor cells in comparison to plasma; however, we did not explore any concentrative transport mechanisms. For *in vitro* studies, we utilized 10- and 20-mM concentrations of ketone bodies that are higher than physiological levels in plasma, but these are somewhat comparable to the concentrations in tumor cells *in vivo* (ranging from 0.85 to 7.84 mM; average 2.41 mM), in mice fed with a ketogenic diet. Furthermore, using similar concentrations, we observed no significant alteration in viability of non-transformed pancreatic ductal epithelial cells (Additional file 2). It indicates that the utilized doses do not affect non-cancer cell survival. In addition, our experiments indicate that ketone bodies do get metabolized in tumor cells that would further diminish actual concentration of ketone bodies in the tissues. The need for higher levels of ketone bodies in cell culture conditions to have biological efficacy might in part be due to higher glucose levels in the culture conditions (25 mM) than the physiological concentration (5.5 mM; even lower in tumor-bearing mice). Hence, if the effects of ketone bodies are simply due to decreased tumoral glucose levels, it is expected that the cell culture conditions would require higher doses. Regardless, the discrepancy in the levels of ketone bodies required to achieve the biological effects in culture conditions and in mice models reflects the inherent differences in the two experimental systems.

Diet is a key player in the progression and pathogenesis of cancer. While high-calorie and high-fat diets are associated with increased incidence of cancer [59], several epidemiological studies have demonstrated a direct relationship between low-sugar diet and a lower incidence of cancer [60]. Our study indicates a reduced tumor growth and tumor weight, along with a reduced proliferation of tumor cells in tumor-bearing mice that were subjected to a ketogenic diet relative to regular chow. Overall, along with a reduced tumor burden, the ketogenic diet also improved muscle mass and body weight in tumor-bearing mice. Hence, a ketogenic diet may serve as an anticancer agent as well as an anticachectic agent.

## Conclusions

Metabolic alterations, being a hallmark of cancer, can be utilized to target different aspects of tumor growth or associated phenotypes. Cancer cachexia is associated with significant mortality in pancreatic cancer patients. In the present study, we investigated a novel systemic approach to modulate pancreatic cancer cell metabolism for diminishing tumor cell survival and ameliorating cancer cachexia. We have demonstrated anticancerous and anticachectic properties of ketone bodies in cell culture conditions*,* as well as the effect of a ketogenic diet on tumor burden and cachexia in animal models*.* Furthermore, our studies establish a ketone body-induced metabolomics reprogramming as the mechanism of action of a ketogenic diet against cancer and cancer-induced cachexia.

## Abbreviations

BPA: 3-bromopyruvic acid; CCCM: cancer cell-conditioned medium; DG: 2-deoxyglucose; DHE: dihydroethidium; GLUT1: glucose transporter-1; HKII: hexokinase II; HSL: hormone sensitive lipase; HSQC: hetero-nuclear single quantum coherence; IBMX: methylisobutylxanthine; IFN-γ: interferon gamma; IL-6: interleukin-6; LDHA: lactate dehydrogenase A; LiAcAc: lithium acetoacetate; MAFbx: muscle atrophy F-box protein; MTT: 3-[4,5-dimethylthiazol-2-yl]-2,5-diphenyltetrazolium bromide; Murf1: muscle-specific ring finger protein 1; NaHB: 3-hydroxybutyrate; OPLS-DA: orthogonal projections to latent structures discriminant analysis; PDAC: pancreatic ductal adenocarcinoma; ROS: reactive oxygen species; TMSP: 3-(tetramethysilane) propionic acid-2,2,3,3-d_4_; TNFα: tumor necrosis factor alpha; Zag: zinc alpha-2-glycoprotein 1.

## Competing interests

The authors have declared that no competing interests exist.

## Authors’ contributions

SKS designed the experiments; performed experiments with cell culture, animal models, and metabolomics studies; and drafted the manuscript. TG performed metabolomics studies. VP performed the animal studies. NVC carried out cell culture studies. VG performed metabolomics studies. PR assisted with the animal studies. KM conceived the study and helped draft the manuscript. IIP participated in the design of the study and helped draft the manuscript. RP designed and supervised metabolomics studies. FY performed and advised for the statistical analyses. PKS participated in its design, execution, and coordination and helped to draft the manuscript. All authors read and approved the final manuscript.

## Supplementary Material

Additional file 1 **(A) Sequence of qRT-PCR primers. (B)** Composition of ketogenic diet [RESEARCH DIETS D10082502]. Click here for file

Additional file 2 **OPLS-DA analysis. (A)** Validation model of a 1000 random permutation test for the OPLS-DA model. **(B)***RQ* plot for the OPLS-DA model. *R*^2^ = 0.95863, *Q*^2^ = 0.95863. Click here for file

Additional file 3 **Effect of sodium hydroxybutyrate and lithium acetoacetate on non-transformed cells survival. (A)** HPNE and **(B)** RAPAN cells (hTERT-immortalized pancreatic epithelial cell lines) were treated with different concentrations of sodium- 3-hydroxybutyrate (NaHB) and lithium acetoacetate (LiAcAc) for 72 h and cell viability was determined by MTT assay. Click here for file

Additional file 4 **Effect of *S*-hydroxybutyrate and lithium chloride on pancreatic cancer cell survival.** S2-013 **(A)** and Capan 1 **(B)** cells were treated with *S*-hydroxy butyric acid (SHB) and LiCl for 72 h, and cell viability was determined by MTT assay. Click here for file

Additional file 5 **Effect of sodium hydroxybutyrate and lithium acetoacetate on intracellular pH.** S2-013 **(A)**, Capan1 **(B)**, HPNE **(C)**, and RAPAN **(D)** cells were treated with different doses of NaHB, LiAcAc, and SHB for 6 h, and pH was determined by using BCPCF-AM dye. Click here for file

Additional file 6 **Effect of *S*-hydroxy butyric acid and LiCl on gene expression and glucose uptake.(A)** Relative mRNA expression levels of GLUT1, HKII, and LDHA in S2-013 cells treated with 10- and 20-mM concentrations of SHB and LiCl for 24 h. Beta-actin was used as internal control. **(B)** S2-013 cells were treated with 10- and 20-mM concentrations of SHB and LiCl for 24 h, and glucose uptake was measured by using 3H-2DG uptake. Click here for file

Additional file 7 **Backscale plot generated from OPLS-DA scores.** The red color peaks are spectral regions that have higher contribution for class separation. The positive and negative peaks represent metabolites that increased in S2-013 cells and S2-013 cells treated with 20 mM NaHB, respectively. Click here for file

Additional file 8 **Ketone bodies are metabolized by pancreatic cancer cells.** 2D ^1^H-^13^C HSQC spectrum collected on the metabolome extracted from S2-013 cells after treatment with 20 mM ^13^C_4_-labeled 3-hydroxybutyrate (3-HB). Click here for file

Additional file 9 **Metabolic inhibition in tumor cells and cachectic marker expression in myotubes and adipocytes. (A)** S2-013 cells were treated with different doses of BPA for 24 h, and glucose uptake was determined by performing 3H-2DG uptake assay. **(B)** Glucose uptake was determined by performing 3H-2DG uptake assays in S2-013-shScr and S2-013-sh*GLUT1* after 24 h of seeding. S2-013 cells were treated with solvent control, 20 mM NaHB (NaHB-S2-013), and 20 mM LiAcAc (LiAcAc-S2-013) for 24 h. The cells were then washed twice with phosphate-buffered saline and cultured in serum-free DMEM. After 24 h, the conditioned medium was collected. **(C)** Differentiated 3T3L1 cells were cultured in the mentioned conditions for 24 h along with lactate (2 mM) and alanine (2 mM). Total RNA was isolated and relative mRNA levels of *HSL* and *Zag* were determined by qRT-PCR. β-Actin was utilized as an internal control. **(D)** Differentiated myotube form C2C12 cells were cultured in similar conditions for 24 h along with lactate (2 mM) and alanine (2 mM). Total RNA was isolated and relative mRNA levels of *MuRF1* and *Atrogin* were determined by qRT-PCR. β-Actin was utilized as an internal control. Click here for file

Additional file 10 **3-Hydroxybutyrate levels in plasma and tumor specimens from ketogenic diet-fed mice.** Plasma and tumor tissue metabolites were extracted by methanol extraction and the levels of 3-hydroxybutyrate were determined by NMR analysis. The exact concentrations were calculated by generating a standard curve for multiple concentrations of 3-hydroxybutyrate. Click here for file

Additional file 11 **Representative 1H NMR spectra for 140 mg of tumor extracts from normal diet- and ketogenic diet-fed mice.** Tumors were homogenized and metabolites were extracted using 80% cryogenically cold methanol and water. Peak represents methyl peaks of 3-hydroxybutyrate. Dashed line represents 3-hydroxybutyrate in ketogenic diet-fed mice and solid line represents the same from normal diet-fed mice. Click here for file
