# Correction: Metabolic reprogramming induced by ketone bodies diminishes pancreatic cancer cachexia

**DOI:** 10.1186/2049-3002-2-22

**Published:** 2014-09-29

**Authors:** Surendra K Shukla, Teklab Gebregiworgis, Vinee Purohit, Nina V Chaika, Venugopal Gunda, Prakash Radhakrishnan, Kamiya Mehla, Iraklis I Pipinos, Robert Powers, Fang Yu, Pankaj K Singh

**Affiliations:** 1 The Eppley Institute for Research in Cancer and Allied Diseases, University of Nebraska Medical Center, Omaha, NE 68198, USA; 2 Department of Chemistry, University of Nebraska—Lincoln, Lincoln, NE 68588, USA; 3 Department of Pathology and Microbiology, University of Nebraska Medical Center, Omaha, NE 68198, USA; 4 Department of Cellular and Integrative Physiology, University of Nebraska Medical Center, Omaha, NE 68198, USA; 5 Department of Surgery, University of Nebraska Medical Center, Omaha, NE 68198, USA; 6 Department of Biostatistics, University of Nebraska Medical Center, Omaha, NE 68198, USA; 7 Department of Biochemistry and Molecular Biology, University of Nebraska Medical Center, Omaha, NE 68198, USA; 8 Department of Genetic Cell Biology and Anatomy, University of Nebraska Medical Center, Omaha, NE 68198, USA

## Correction

After publication of this Research Article [1], we noticed we had included an incorrect image in Figure 1D in the panel for LiAcAc 20 mM. A corrected Figure 1 is included here.

**Figure 1:**
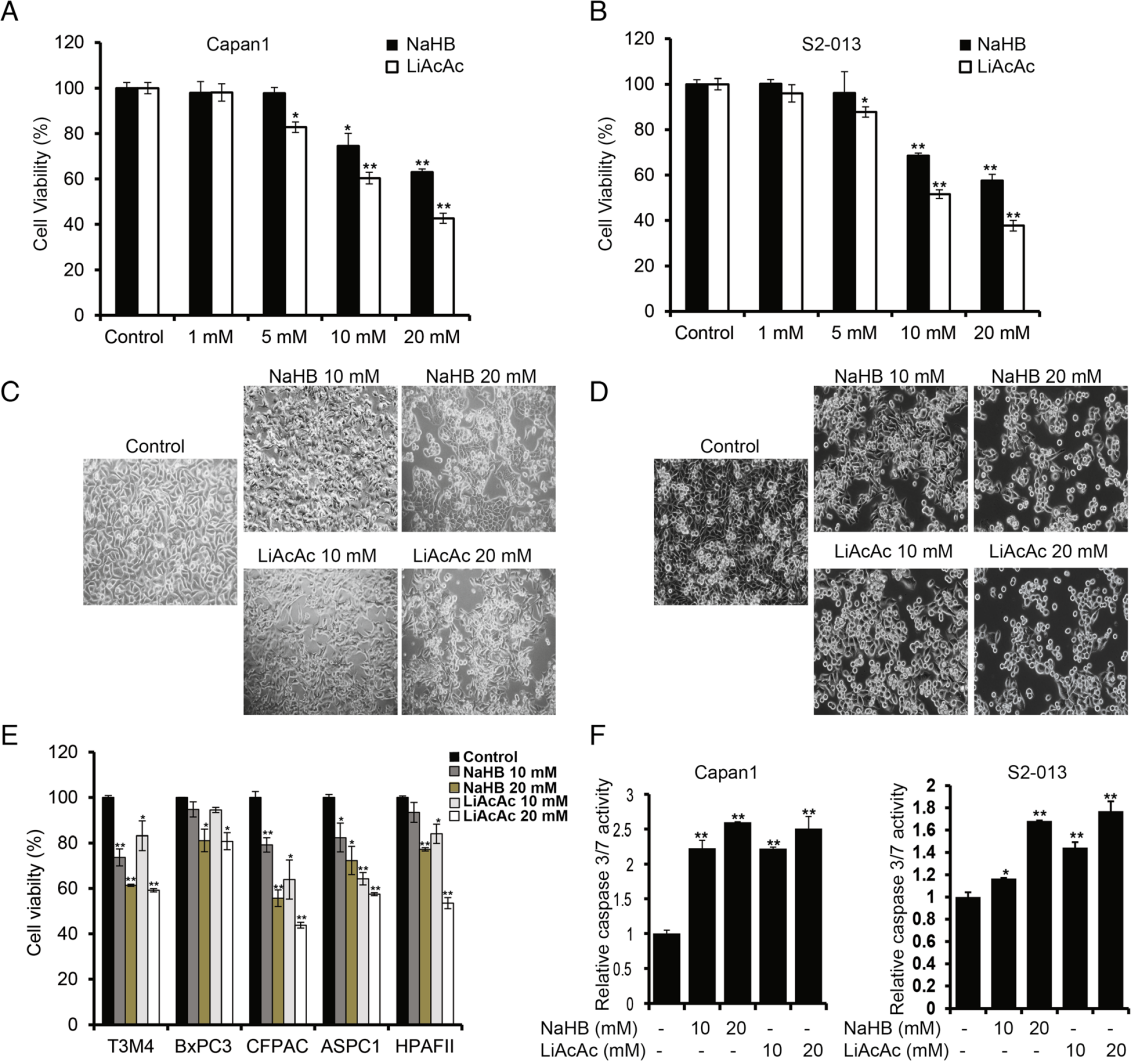
**Ketone bodies inhibit growth and induce apoptosis in pancreatic cancer cell lines.** Capan1 **(A)** and S2-013 **(B)** cells were treated with different concentrations of sodium-3-hydroxybutyrate (NaHB) and lithium acetoacetate (LiAcAc) for 72 h, and cell viability was determined by MTT assay. *Bar* represents percent viability under indicated treatments relative to treatment with solvent control. Representative bright-field images of Capan1 **(C)** and S2-013 **(D)** cells under treatment with 10- and 20-mM concentrations of NaHB and LiAcAc for 72 h. **(E)** Multiple pancreatic cancer cell lines were treated with 10- and 20-mM concentrations of NaHB and LiAcAc for 72 h, and relative cell viability determined by MTT assay is plotted in the *bar charts*. **(F)** Capan1 and S2-013 cells treated with 10- and 20-mM concentrations of sodium-3-hydroxybutyrate and lithium acetoacetate for 48 h and the relative caspase 3/7 activity are plotted. Values represented are mean ± SEM. *P<0.05; **P<0.01.
