# Supplementary figures and images for: Metabolic reprogramming induced by ketone bodies diminishes pancreatic cancer cachexia
Source: Cancer Metab. 2014 Sep 1;2:18. doi: 10.1186/2049-3002-2-18 (PMC4165433; doi:10.1186/2049-3002-2-18)

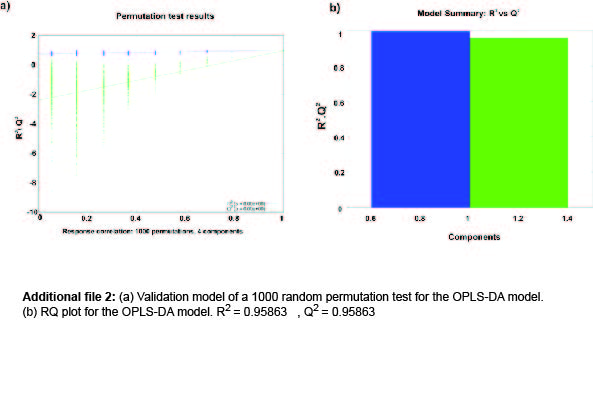

Supplement: Additional file 2 — OPLS-DA analysis. (A) Validation model of a 1000 random permutation test for the OPLS-DA model. (B)RQ plot for the OPLS-DA model. R2 = 0.95863, Q2 = 0.95863. [file 2049-3002-2-18-S2.jpeg]

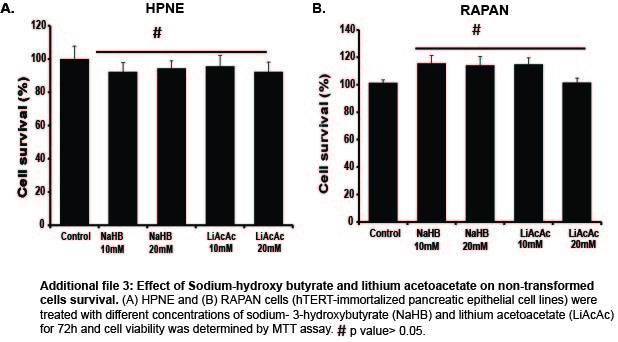

Supplement: Additional file 3 — Effect of sodium hydroxybutyrate and lithium acetoacetate on non-transformed cells survival. (A) HPNE and (B) RAPAN cells (hTERT-immortalized pancreatic epithelial cell lines) were treated with different concentrations of sodium- 3-hydroxybutyrate (NaHB) and lithium acetoacetate (LiAcAc) for 72 h and cell viability was determined by MTT assay. [file 2049-3002-2-18-S3.jpeg]

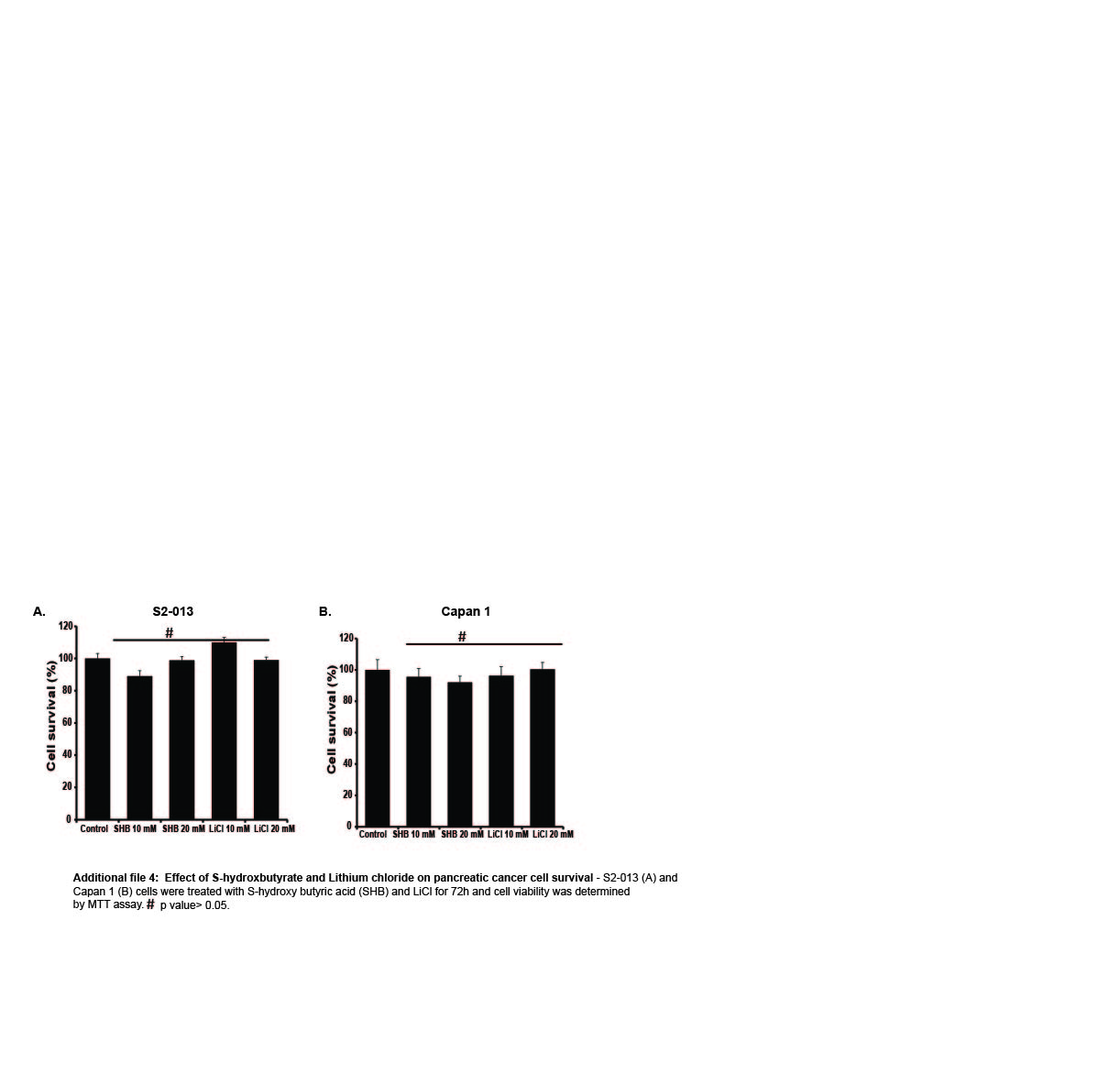

Supplement: Additional file 4 — Effect of S -hydroxybutyrate and lithium chloride on pancreatic cancer cell survival. S2-013 (A) and Capan 1 (B) cells were treated with S-hydroxy butyric acid (SHB) and LiCl for 72 h, and cell viability was determined by MTT assay. [file 2049-3002-2-18-S4.jpeg]

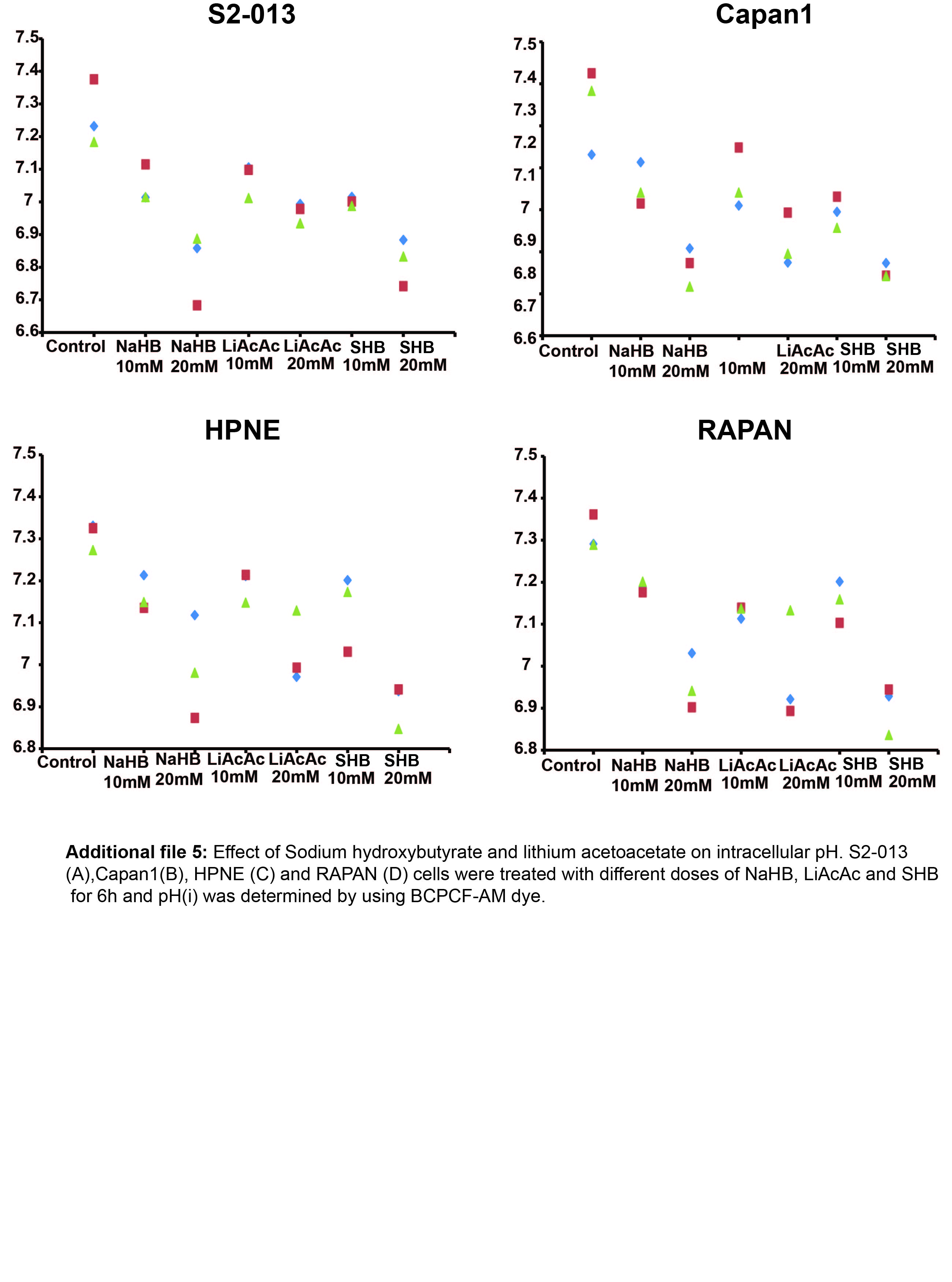

Supplement: Additional file 5 — Effect of sodium hydroxybutyrate and lithium acetoacetate on intracellular pH. S2-013 (A), Capan1 (B), HPNE (C), and RAPAN (D) cells were treated with different doses of NaHB, LiAcAc, and SHB for 6 h, and pH was determined by using BCPCF-AM dye. [file 2049-3002-2-18-S5.jpeg]

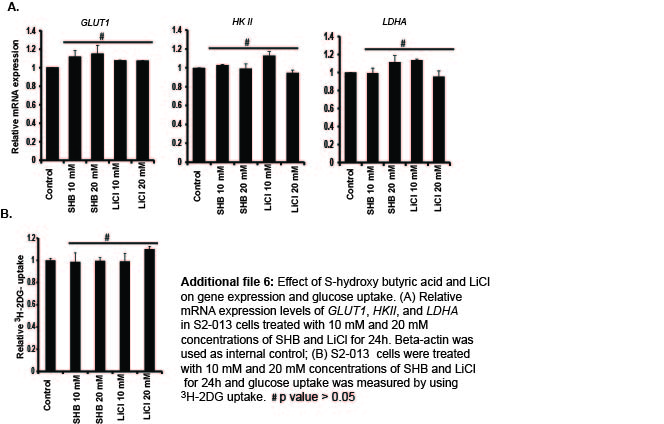

Supplement: Additional file 6 — Effect of S -hydroxy butyric acid and LiCl on gene expression and glucose uptake.(A) Relative mRNA expression levels of GLUT1, HKII, and LDHA in S2-013 cells treated with 10- and 20-mM concentrations of SHB and LiCl for 24 h. Beta-actin was used as internal control. (B) S2-013 cells were treated with 10- and 20-mM concentrations of SHB and LiCl for 24 h, and glucose uptake was measured by using 3H-2DG uptake. [file 2049-3002-2-18-S6.jpeg]

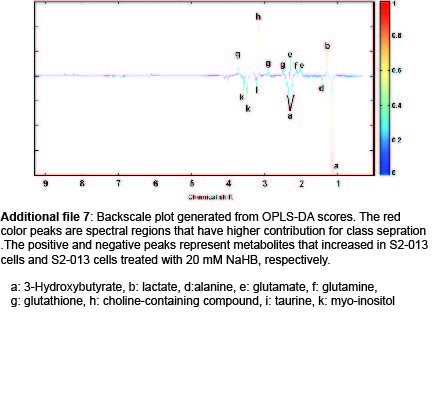

Supplement: Additional file 7 — Backscale plot generated from OPLS-DA scores. The red color peaks are spectral regions that have higher contribution for class separation. The positive and negative peaks represent metabolites that increased in S2-013 cells and S2-013 cells treated with 20 mM NaHB, respectively. [file 2049-3002-2-18-S7.jpeg]

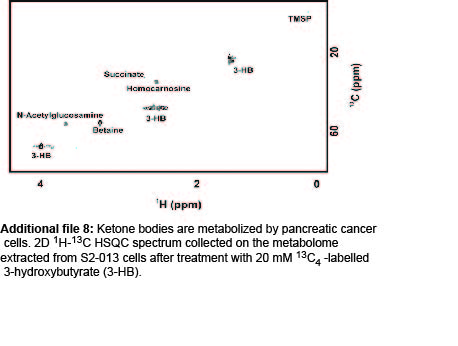

Supplement: Additional file 8 — Ketone bodies are metabolized by pancreatic cancer cells. 2D 1H-13C HSQC spectrum collected on the metabolome extracted from S2-013 cells after treatment with 20 mM 13C4-labeled 3-hydroxybutyrate (3-HB). [file 2049-3002-2-18-S8.jpeg]

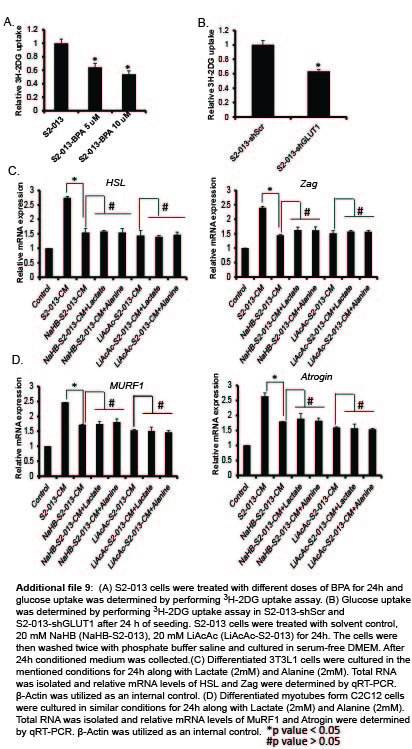

Supplement: Additional file 9 — Metabolic inhibition in tumor cells and cachectic marker expression in myotubes and adipocytes. (A) S2-013 cells were treated with different doses of BPA for 24 h, and glucose uptake was determined by performing 3H-2DG uptake assay. (B) Glucose uptake was determined by performing 3H-2DG uptake assays in S2-013-shScr and S2-013-shGLUT1 after 24 h of seeding. S2-013 cells were treated with solvent control, 20 mM NaHB (NaHB-S2-013), and 20 mM LiAcAc (LiAcAc-S2-013) for 24 h. The cells were then washed twice with phosphate-buffered saline and cultured in serum-free DMEM. After 24 h, the conditioned medium was collected. (C) Differentiated 3T3L1 cells were cultured in the mentioned conditions for 24 h along with lactate (2 mM) and alanine (2 mM). Total RNA was isolated and relative mRNA levels of HSL and Zag were determined by qRT-PCR. β-Actin was utilized as an internal control. (D) Differentiated myotube form C2C12 cells were cultured in similar conditions for 24 h along with lactate (2 mM) and alanine (2 mM). Total RNA was isolated and relative mRNA levels of MuRF1 and Atrogin were determined by qRT-PCR. β-Actin was utilized as an internal control. [file 2049-3002-2-18-S9.jpeg]

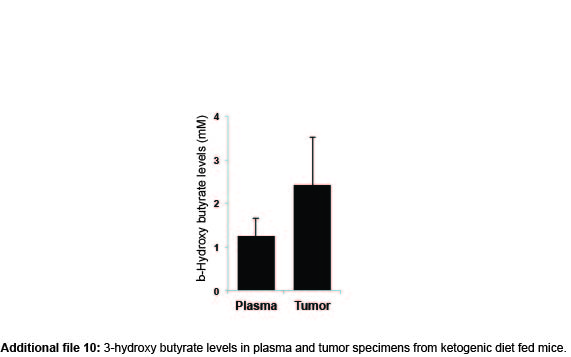

Supplement: Additional file 10 — 3-Hydroxybutyrate levels in plasma and tumor specimens from ketogenic diet-fed mice. Plasma and tumor tissue metabolites were extracted by methanol extraction and the levels of 3-hydroxybutyrate were determined by NMR analysis. The exact concentrations were calculated by generating a standard curve for multiple concentrations of 3-hydroxybutyrate. [file 2049-3002-2-18-S10.jpeg]

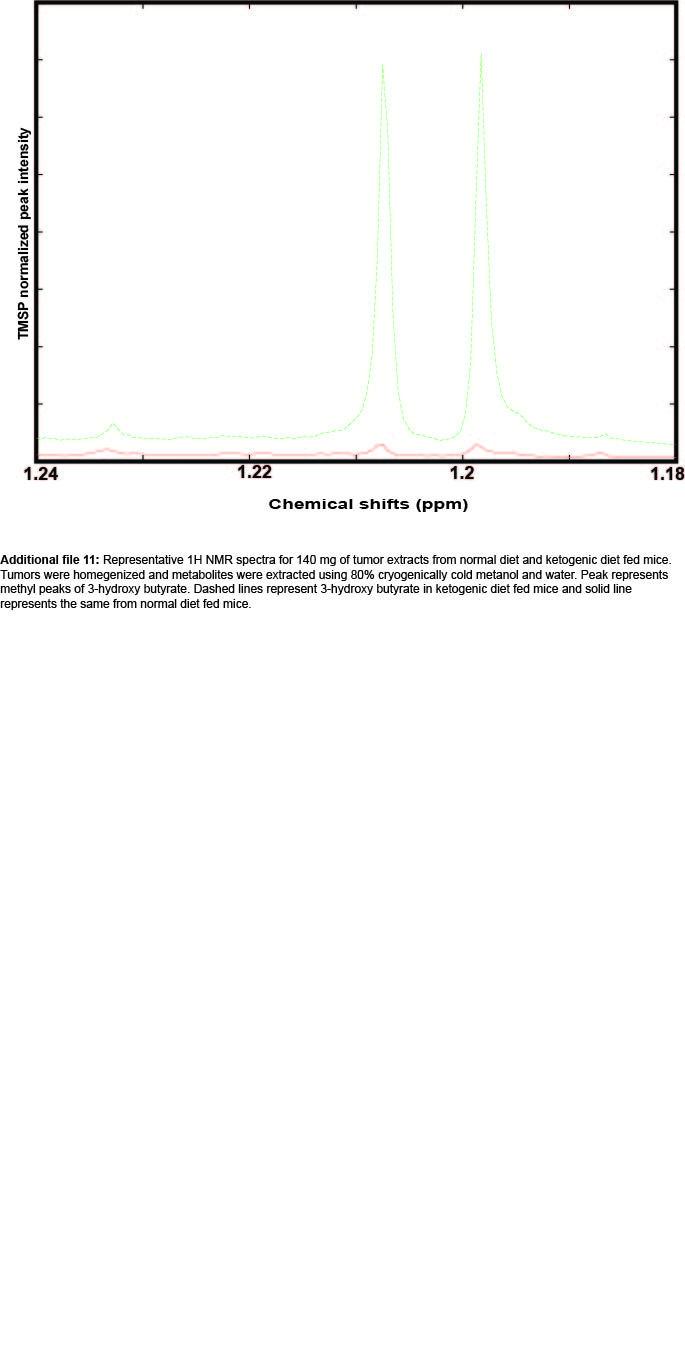

Supplement: Additional file 11 — Representative 1H NMR spectra for 140 mg of tumor extracts from normal diet- and ketogenic diet-fed mice. Tumors were homogenized and metabolites were extracted using 80% cryogenically cold methanol and water. Peak represents methyl peaks of 3-hydroxybutyrate. Dashed line represents 3-hydroxybutyrate in ketogenic diet-fed mice and solid line represents the same from normal diet-fed mice. [file 2049-3002-2-18-S11.jpeg]
