# Supplementary material for: Metabolic reprogramming induced by ketone bodies diminishes pancreatic cancer cachexia
Source: Cancer Metab. 2014 Sep 1;2:18. doi: 10.1186/2049-3002-2-18 (PMC4165433; doi:10.1186/2049-3002-2-18)
Supplement: Supplementary file 2 [file 2049-3002-2-18-S1.doc]

Additional file 1

A. Sequence of qRT-PCR primers.

Gene	Sequence (5'-3')
hGLUT1-F	GATTGGCTCCTTCTCTGTGG
hGLUT1-R	TCAAAGGACTTGCCCAGTTT
hHKII-F	GAGCCACCACTCACCCTACT
hHKII-R	ACCCAAAGCACACACGGAAGTT
hLDHA-F	TTGACCTACGTGGCTTGGAAG
hLDHA-R	GGTAACGGAATCGGGCTGAAT
mMuRF1-F	AGGACAACCTCGTGCCTACAAG
mMuRF1-R	ACAACCTGTGCCGCAAGTG
mAtrogin-F	TGAGCGACCTCAGCAGTTAC
mAtrogin-R	GCGCTCCTTCGTACTTCCTT
mZAG-F	GAGCCTGTGGGACCTTGGA
mZAG-R	CCTCCCTGGCCCTCTGAA
mHSL-F	GGCTTACTGGGCACAGATACCT
mHSL-R	CTGAAGGCTCTGAGTTGCTCAA
ch-myc-hGLUT1-F	CTGCTCACTCATTCGTGCAT
ch-myc-hGLUT1-R	TGCCACTTGTCCTCTGTCTG
ch-myc-hLDHA-F	CGACTCACACGTGGGTTCC
ch-myc-hLDHA-R	GGGCCTTAAGTGGAACAGC

B. Composition of ketogenic-diet [RESEARCH DIETS D10082502]

Component	gm%	Kcal%
Protein	28	18
Carbohydrate	1	1
Fat	58	81
**Ingredient**	**gm**	**kcal**
Casein	195	780
DL-Methionine	3	12
Corn Starch	0	0
Maltodextrin	0	0
Sucrose	0	0
Cellulose, BW200	50	0
Milk fat, Anhydrous	194	1476
Lard	19.4	175
Corn oil	35	0
Mineral mixS10001	4	0
Calcium Carbonate	10	40
Vitamin Mix V10001	2	0
Choline Bitartrate	2	0
Cholestrol	1.5	0
Ethoxyquin	0.5	0
